# A platform for modular assembly and feeding of micro-organoids on standard Petri dishes

**DOI:** 10.1242/bio.059825

**Published:** 2023-05-19

**Authors:** Federico Nebuloni, Joseph Morgan, Edmond J. Walsh, Peter R. Cook

**Affiliations:** ^1^ Osney Thermofluids Institute, Department of Engineering Science, University of Oxford, Osney Mead, Oxford OX2 0ES, UK; ^2^ The Sir William Dunn School of Pathology, University of Oxford, South Parks Road, Oxford OX1 3RE, UK

**Keywords:** 3D cell culture, FC-40, Fluid wall, Fluorocarbon, Organoid

## Abstract

Organoids grow *in vitro* to reproduce structures and functions of corresponding organs *in vivo*. As diffusion delivers nutrients over only ∼200 µm, refreshing flows through organoids are required to avoid necrosis at their cores; achieving this is a central challenge in the field. Our general aim is to develop a platform for culturing micro-organoids fed by appropriate flows that is accessible to bioscientists. As organs develop from layers of several cell types, our strategy is to seed different cells in thin modules (i.e. extra-cellular matrices in stronger scaffolds) in standard Petri dishes, stack modules in the required order, and overlay an immiscible fluorocarbon (FC40) to prevent evaporation. As FC40 is denser than medium, one might expect medium to float on FC40, but interfacial forces can be stronger than buoyancy ones; then, stacks remain attached to the bottom of dishes. After manually pipetting medium into the base of stacks, refreshing upward flows occur automatically (without the need for external pumps), driven mainly by differences in hydrostatic pressure. Proof-of-concept experiments show that such flows support clonal growth of human embryonic kidney cells at expected rates, even though cells may lie hundreds of microns away from surrounding fluid walls of the two immiscible liquids.

## Teaser

**Summary:** Refreshing flows through organoids are required to avoid necrosis at their cores; we develop a platform for culturing micro-organoids fed by appropriate flows that is accessible to bioscientists.

## INTRODUCTION

One dream of current biomedicine is to generate functional organs *in vitro* that can replace their faulty counterparts *in vivo* (Simian and Bissell, 2017; Tuveson and Clevers, 2019). Various approaches are being actively prosecuted: for example, individual cell types are grown separately *in vitro* and assembled into organ-like structures, and stem cells are cultured *in vitro* under conditions that recapitulate development *in vivo* (Simian and Bissell, 2017; Tuveson and Clevers, 2019; Paşca et al., 2022). As miniaturization brings many advantages, ‘organs-on-a-chip’ are also being built to realize these approaches (Park et al., 2019; Sonnen and Merten, 2019; Dudukovic et al., 2021; Vunjak-Novakovic et al., 2021). However, take-up of associated devices by the biomedical community has not fulfilled initial hopes, for reasons that include: devices being made with materials unfamiliar to biologists; devices not fitting easily into their workflows; devices usually being dedicated to one application; prototyping taking days and even weeks; operations requiring specialized equipment and training; and solid confining walls limiting access (Sackmann et al., 2014; Ortseifen et al., 2020).

We recently introduced ways of building microfluidic devices in which small volumes are confined by liquid walls rather than solid ones; these walls are easily pierced by pipettes at any point (Walsh et al., 2017; Soitu et al., 2018, 2020b; Deroy et al., 2021a). In this form of open microfluidics (Berthier et al., 2019), fluid walls are created in seconds on standard Petri dishes by reshaping two immiscible liquids (usually cell-growth media and the fluorocarbon, FC40); then, the aqueous phase is pinned to the dish by interfacial forces. These walls have many properties useful for both microfluidic and biomedical applications (Riess and Krafft, 1998; Krafft and Reiss, 2015; Deroy et al., 2021a). They limit aqueous evaporation (the solubility of water in FC40 is <7 ppm by weight at room temperature, compared to <200 ppm for the silicone oil widely used by bioscientists), while providing a sterility barrier between cells and their surroundings (Soitu et al., 2018). They are made of a material not found in nature that is arguably the most bioinert liquid known, and which is so permeable to O_2_ and CO_2_ that close relatives have been used as blood substitutes (Sarkar et al., 2014). These walls are also transparent with a refractive index of 1.29 close to the 1.33 of water, so one can see everything behind them with little diffractive distortion – especially useful during microscopic examination (Soitu et al., 2020a). Additionally, FC40 flows much like water (dynamic viscosities at 25°C of water, FC40, silicone oils, and honey are 0.89, 4.1, 2-200, and >2000 cP, respectively).

Thus far, cells have been grown in fluid-walled circuits on the two-dimensional (2D) surface of polystyrene Petri dishes (Deroy et al., 2021a); we now establish methods for three-dimensional (3D) culture. We first list properties one would hope to find in any widely used approach for growing micro-organoids. To build this list, we consider the physical constraints acting during embryogenesis. First, diffusion delivers nutrients to (and removes waste from) cells over only ∼200 µm (Crick, 1970; Sharpe, 2019), so growth beyond the ∼400 µm sphere resulting from a fertilized egg requires refreshing flows through the center. This is established by various mechanisms beginning with self-induced hydraulic fracturing to generate inter-cell micro-bubbles that coalesce to form the blastocoel (Dumortier et al., 2019). Subsequently, cilia drive circulation in this and other cavities (e.g. to create left–right asymmetry; Yoshiba and Hamada, 2014). Only later does an extra-embryonic vasculature form (when the human embryo is ∼4 mm in diameter), and an intra-embryonic one later still. Thereafter, essentially all cells lie within diffusional range of refreshing flows through arterioles and venules (Lee et al., 2018; Wimmer et al., 2019). This points to the critical importance of ensuring flows through cell masses >200 µm thick to avoid necrosis at the core – a central problem during organoid culture (Lancaster and Knoblich, 2014; Park et al., 2019). Second, organs develop from layers of different cell types (Goodwin and Nelson, 2021); for example, appearance of ectoderm, mesoderm and endoderm are the first signs of differentiation after fertilization, and skin (the largest adult organ) consists of layered epidermal, dermal, and fat cells. Third, inter-layer signaling drives subsequent development (Goodwin and Nelson, 2021); it also occurs over <200 µm as it often depends on diffusion. Fourth, cells frequently move over/through adjacent layers (e.g. as neural tubes and cerebral cortex form; Goodwin and Nelson, 2021). Fifth, cells grow in layer-specific extracellular matrices (ECMs) with interconnected pores (∼200–800 µm diameter; Caliari and Burdick, 2016). ECMs can occupy surprisingly large tissue volumes (e.g. 5%, 35%, and 80% in brain, myocardium, and some tumors; Magzoub et al., 2008; Syková and Nicholson, 2008), and cells have even been cultured in 3D in stainless-steel wire meshes with layers spaced ∼100 µm apart (Sakaguchi et al., 2021). Additional attractive features for any platform include the use of small volumes (given the expense of growth factors required to promote cell differentiation *in vitro*), and then organs of >200 µm must be assembled by tessellating smaller pieces (which we will call modules). Moreover, prototyping should be rapid with, for example, modules being easily swapped in and out to test new conditions. Finally, the method should be accessible to bioscientists – which probably means organoids sit in standard Petri dishes where they can be fed with fresh media delivered through standard pipettes and imaged using microscopes found in biolabs.

We now describe a platform incorporating properties in this wish-list (Table S1); it is inspired by an approach that uses solid walls but lacks many such properties (Yu et al., 2019). Our purpose here is to establish physical conditions supporting rapid cell growth of one cell type in standard dishes, and we hope to extend this platform to organoids.

## RESULTS

### Approach

**Fig. 1.**
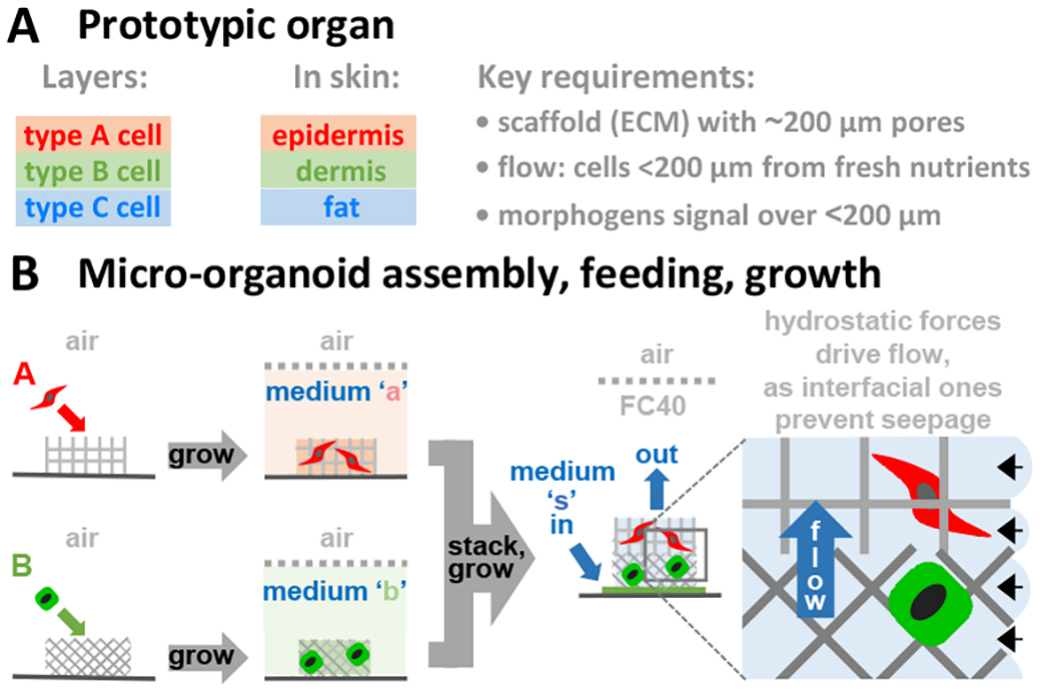
**Overview.** (A) The prototypic organ consists of layers of different cells, with skin being an example; some key requirements for organ culture are listed. (B) General workflow for constructing layers of different cell types, and feeding them in standard dishes and CO_2_ incubators to promote micro-organoid development. Cells A and B are seeded in porous modules containing appropriate ECMs, and grown conventionally in medium ‘a’ or ‘b’. Next, modules are stacked on a filter-paper base (green) sitting in a Petri dish, and overlaid with FC40 to prevent evaporation. After pipetting stacking medium (‘s’) on to the filter paper, refreshing flows up the stack occur in ways to be described. The zoom highlights aqueous continuity between layers, upward flow, and Laplace pressure at FC40:medium interfaces (black arrows) preventing seepage from pores.

Our approach is based on the recognition that organs develop *in vivo* from layers of different cell types under critical length constraints (Fig. 1A). Each layer is held in a module that can be picked up using tweezers without breakage. Many suitable modules are available (Caliari and Burdick, 2016). For example, ones for epidermal and dermal cells can be derived by polymerizing pure collagen or by removing cells from pig skin to leave a complex ECM; both approaches yield dry paper-like aerogels that are easily cut into any desired 2D shape. In a general workflow (Fig. 1B), cell types A and B are pipetted into modules A and B sitting in a standard Petri dish; each module contains an appropriate ECM and is generally <400 µm thick so cells in the center are within diffusional range of the exterior. Filled modules are grown separately and conventionally immersed in their own medium (‘a’ or ‘b’), before they are stacked in the desired order so that aqueous continuity between them is established. FC40 is now overlaid to prevent evaporation, and refreshing flows (here, of stacking medium, ‘s’) are created in ways to be described. Our purpose is to validate steps in this workflow in the hope they might eventually facilitate assembly of micro-organoids into organs by tessellating appropriately-shaped modules.

### Some forces shaping liquid structures

Interfacial and hydrostatic forces play critical roles in this workflow. For example, interfacial forces drive initial cell wicking and dispersal throughout modules (Kumar et al., 2019). They also firmly attach media within modules to dishes so that when FC40 is overlaid – which is ∼1.8 times denser than water – the aqueous phase remains stuck to the dish (providing its volume is small, and its interface with the dish is large). This is counter-intuitive: one might expect less-dense medium to float on the denser fluorocarbon, but interfacial forces between dish and medium can be stronger than buoyancy ones. Where volumes are large and contact areas with the dish small, weights (e.g. stainless-steel washers, nuts) are added on top of modules to prevent floatation.

**Fig. 2.**
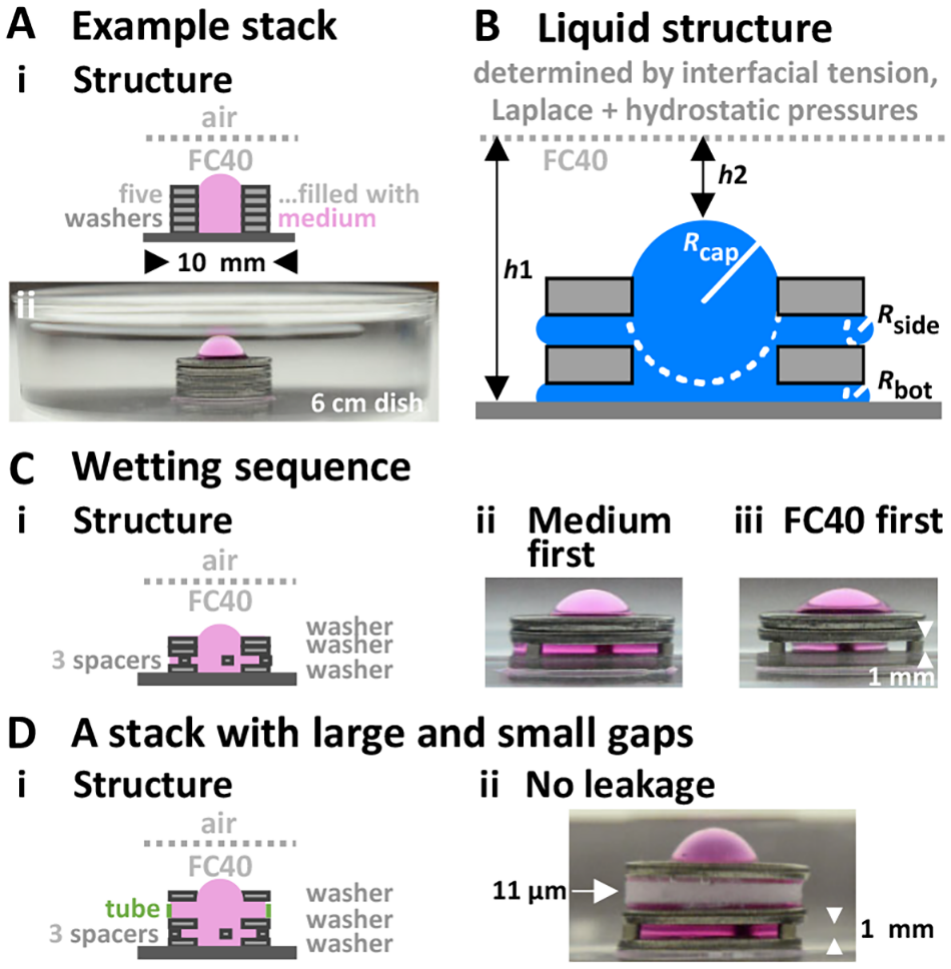
**Example stacks built using stainless-steel washers (OD 10 mm, ID 5 mm, thickness 1 mm) in standard 60 mm dishes.** Medium is retained in all stacks. FC40 is also transparent and invisible in all images that are shown here and subsequently. (A) Stack of five washers. (i) Structure. (ii) Image. (B) Aqueous structure in two-washer stack. Medium is less dense than FC40, so one might expect it to float on the fluorocarbon; however, it remains stuck to the bottom because interfacial forces between polystyrene and water are stronger than buoyancy ones. Interfacial forces also minimize contact between water and FC40 (so interfaces are shaped like spherical caps). Laplace pressure prevents water from seeping out under/between washers (as *R*_bottom_ and *R*_side_ are small). (C) Wetting sequence. (i) Structure (three washers+three spacers – stainless-steel tube magnets, diameter 1 mm, height 1 mm) and (ii) image after filling stack in air with 175 µl medium, and overlaying FC40. (iii) Image after filling the bottom washer in air with 20 µl medium, overlaying FC40, and filling the stack by adding medium through FC40 to that already on the bottom. Additional medium fails to displace FC40 pre-wetting steel (giving a narrower aqueous column). (D) Stack with large and small gaps (of 1 mm due to magnetic spacers, and down to ∼11 µm pores in a filter-paper tube that is 2 mm high). (i) Structure. (ii) Image.

To illustrate the interplay between forces that are exploited, consider a stack of five stainless-steel washers with outer and inner diameters (ODs and IDs) of 10 and 5 mm (more relevant modules are used later). The stack is built manually by placing one washer on top of another in a standard polystyrene Petri dish, and medium pipetted into the middle of the stack. As these washers are not particularly flat or smooth, some medium seeps under the bottom washer and between others; nevertheless, most medium remains confined to the stack, held there by interfacial forces that are strong enough to prevent spreading throughout the dish. When FC40 is overlaid, medium remains in the stack (Fig. 2A). The structure of the aqueous tower is then determined by gravitational forces working through the hydrostatic head of pressure, *P*, and interfacial forces working through differential wetting of polystyrene and steel by each liquid plus Laplace pressure. Thus, *P* is given by *ρgh* (where *ρ* is density, *g* the gravitational constant, and *h* the height of liquid), and the pressure difference, Δ*P*, across any spherical medium:FC40 interface is given by the Young-Laplace equation and is 2γ/*R* (where γ is interfacial tension, and *R* is radius of curvature). Consequently, in Fig. 2B, the Δ*P* required to force medium between washers increases as *R*_side_ shrinks, so Laplace pressure prevents seepage from the stack laterally as *R*_bot_ and *R*_side_ are so small.

One striking feature of these stacks is the spherical cap on the aqueous tower (Fig. 2Aii). Its shape is due to minimization of contact area between immiscible liquids. As *R*_cap_>*R*_bot_ and *R*_side_ (Fig. 2B), medium is more likely to be lost from the stack through this cap. The point with the lowest pressure in the aqueous tower is also at the top, so any medium that initially seeped out from under the bottom washer into the dish is sucked back into the tower on adding FC40. In this sense, the aqueous structure is self-healing.

Spacers can be inserted between stacked washers to create 1 mm gaps (Fig. 2Ci,ii), and yet the fluidic structure is stable enough to be carried around a lab. However, stacks with 2 mm gaps must be carried carefully, and ones with 4 mm gaps are unstable; medium spontaneously detaches from the bottom and floats to the surface (Fig. S1A). Fluidic structure also depends on wetting sequence; if internal sides of upper washers/spacers are wetted with FC40 before adding medium, the aqueous tower is thinner as medium cannot displace the FC40 already wetting the steel (compare Fig. 2Cii with iii). Smaller stacks containing 2 µl medium can even be built in individual wells in 96-well microplates (Fig. S1B). These results show that gaps are easily incorporated into stacks to allow insertion of pipette tips, that wetting sequence determines fluidic structures, and there is potential to miniaturize stacks. They are also consistent with many results obtained previously in open microfluidics (Lee et al., 2012; Berthier et al., 2019).

We next build a stack containing openings larger and smaller than ever likely to be needed in our general workflow – one with a 1 mm gap (to allow access of a pipette tip), plus pores down to ∼11 µm (that are smaller than the pores in most ECMs, and the smallest in a 2 mm-high tube made from Whatman number 1 filter paper; Fig. 2D). These results indicate that FC40 walls can contain medium in modules that have gaps/pores with sizes in our wanted range, and that this is easily achieved without special gaskets or seals.

### Driving flow through modules without using external pumps

**Fig. 3.**
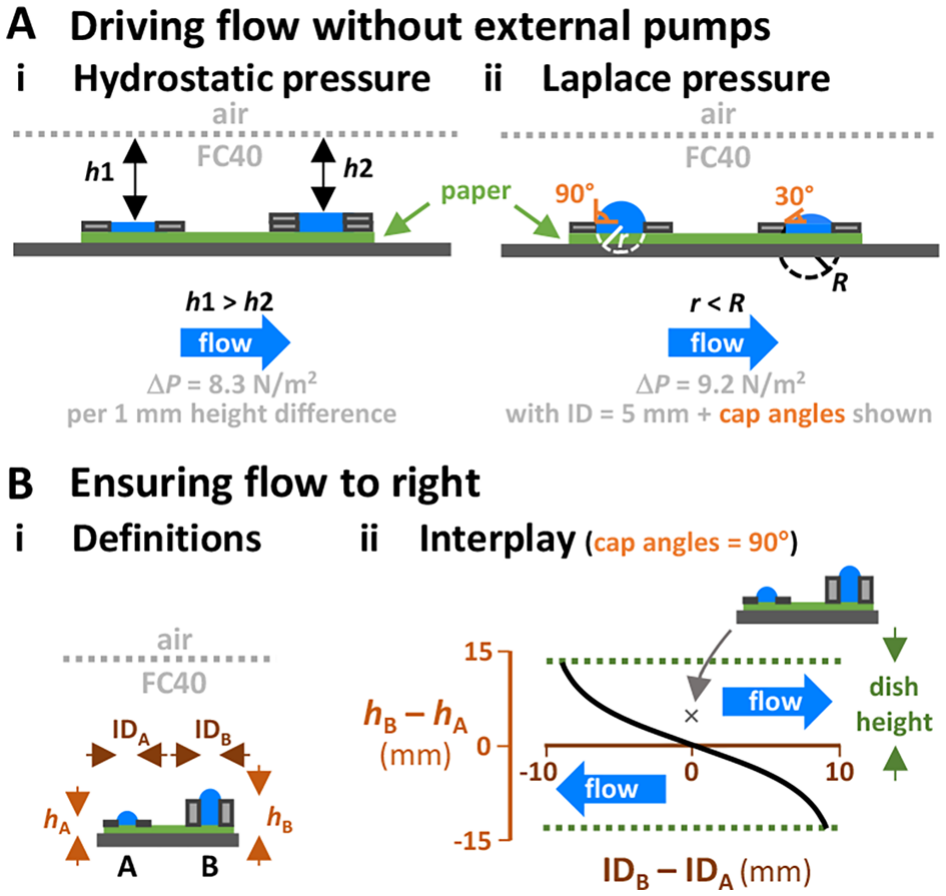
**Driving flow without using external pumps.** (A) Principles. Stacks filled with medium (blue) sit on medium-saturated filter paper (green) under FC40. Δ*P* is the pressure difference. (i) Differences in hydrostatic heads of dense FC40 over stacks drive flow to right (cap radii and angles are identical, *ρ*=1000 kg/m^3^. (ii) Difference in Laplace pressure drive flow to right. Δ*P* is calculated neglecting differences in hydrostatic head due to differences in cap height, and using γ=23 mN/m for DMEM+10% FBS under FC40 (Deroy et al., 2021b). (B) Ensuring flow is to right. (i) Definitions. (ii) Interplay between hydrostatic and Laplace pressures determines flow direction (flow to the right is increased by increasing *h*_B_ relative to *h*_A_, by reducing ID_A_ relative to ID_B_, and by increasing cap angle in stack A relative to that in B by filling caps to different degrees). Dish height (∼13 mm for a 60 mm dish) limits the maximum difference in hydrostatic head possible (dotted lines), and a cap angle of 90° yields the maximum Laplace pressure with a given ID. The black curve (calculated using 90° angles, *ρ*=998 kg/m^3^, and γ=23 mN/m) indicates where differences in hydrostatic and Laplace pressures are equal, and provides a simple approximation for assessing flow direction as stack height and washer ID vary. Flow is to the right above this curve – e.g., at point x (where *h*_A_=1 mm, *h*_B_=5 mm, ID_A_=ID_B_=5 mm; the cartoon illustrates this structure).

Feeding organoids presents a central challenge in the field (Lancaster and Knoblich, 2014; Park et al., 2019). Syringe pumps are widely used to drive flows, but are not designed to operate in humid CO_2_ incubators; bioscientists unfamiliar with microfluidic techniques also find it difficult to use them, especially in shared incubators. As is well known in the field of microfluidics, flows are easily generated without external pumps. Thus, in Fig. 3Ai, *h*1 is larger than *h*2, so a greater hydrostatic head of dense FC40 over the left-hand stack can drive medium to the right. Flow also occurs between identical stacks if their spherical caps have different radii of curvature and so Laplace pressures (Fig. 3Aii, *r*<*R*; MacLeod and Radke, 1993; Walker and Beebe, 2002). Consequently, differences in hydrostatic head and radius of curvature both influence flow direction. To ensure flow is in the desired direction, we added extra washers to recipient stacks to create differences in hydrostatic head large enough to overcome opposing differences in Laplace pressure. Fig. 3B illustrates how to achieve this with stacks in 60 mm dishes (for the underlying theory, see Fig. S2 and Materials and Methods). From now on, flow through circuits will mainly be driven by differences in hydrostatic pressure.

**Fig. 4.**
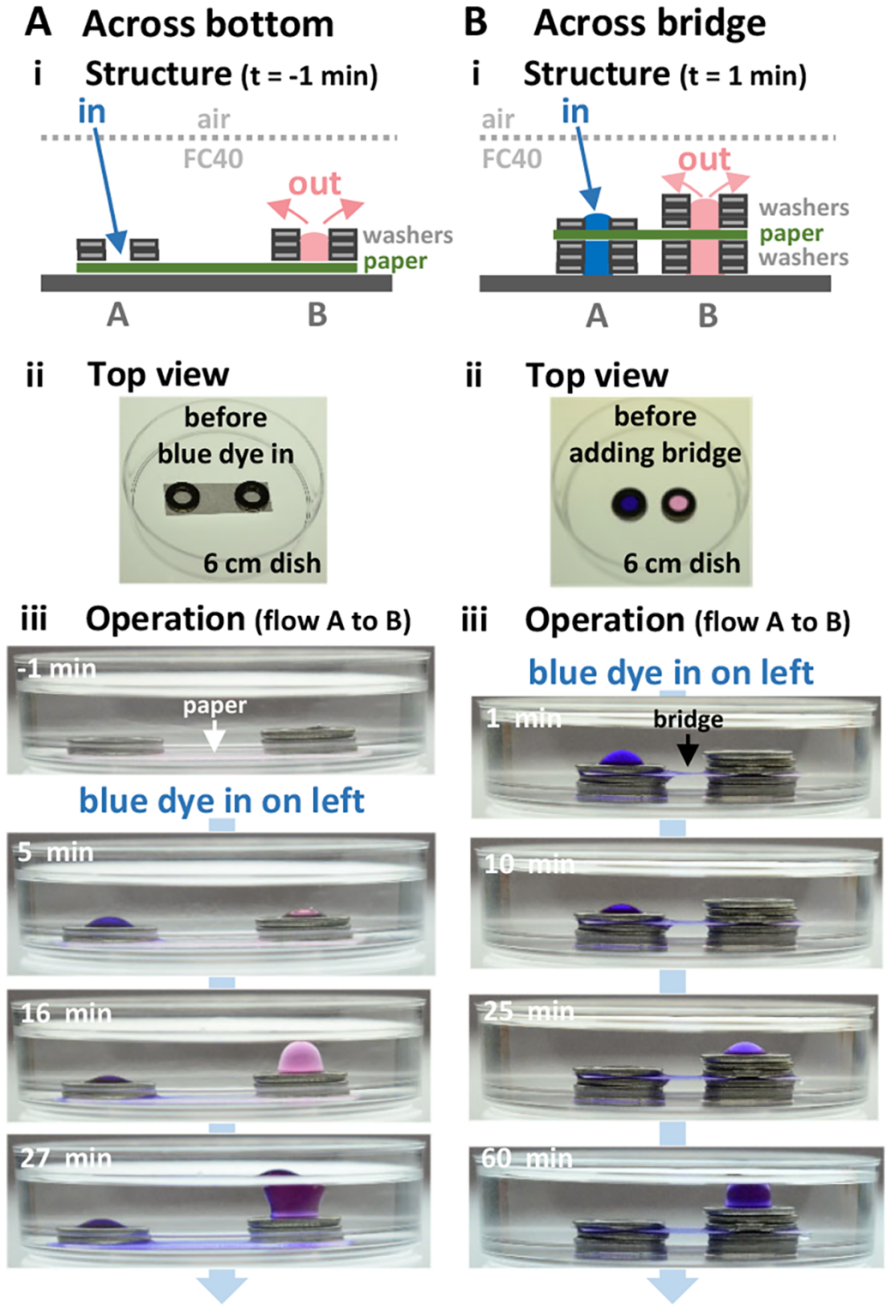
**Driving flow between stacks of washers (OD 10 mm, ID 5 mm, hole volume** ∼**20 µl) through filter paper (without using external pumps).** (A) Flow on bottom. (i) Structure. (ii) Image of stacks sitting in air on dry paper (30×10 mm) before adding medium, FC40, or blue dye. (iii) Images after adding medium to wet the filter paper, filling stack B with more medium than A, and quickly overlaying FC40. Now (at t=−1 min), medium has accumulated in stack B. At t=0 min, medium+blue dye is pipetted into stack A through FC40 on to the filter paper (50 µl added at t=0 and 5 min, and thereafter 25 µl every 5 min for 25 min). Dye flows from stack A through the paper to the cap on B (giving purple). (B) Flow through bridge. (i) Structure. Note there is no paper base. (ii) Image of two three-washer stacks before building bridge. The structure is completed by filling stack A with medium+blue dye and stack B with medium, overlaying a paper bridge (25×10 mm) wetted with medium, adding one and three washers, and overlaying FC40. (iii) Images at different times after repeatedly adding 25 µl medium+blue dye every ∼6 min through FC40 on to the filter paper in stack A. Dye flows through the paper bridge to the cap on B.

We next demonstrated the principles driving flows in practice. We built two stacks of washers sitting on a dry filter paper (Whatman number 1, thickness ∼180 µm) in air, wet the paper with medium, fill stacks, and overlay FC40; interfacial forces hold medium in the circuit (Fig. 4Ai,ii). When we manually pipetted medium plus blue dye at intervals into stack A through FC40 on to the paper, interfacial forces held the added medium to the pre-wetted paper. Differences in hydrostatic pressure now become the main drivers of flow into the cap on stack B (Fig. 4Aiii). As stacks are ∼3 cm apart, diffusion is too slow to play a significant role in this dye transfer (Fig. S3A; Fan et al., 2018). As more dye is added, the right-hand cap enlarges, balloons up, buds off, and finally floats on FC40 (Fig. S3B). Such automatic self-emptying will be exploited when feeding for long periods. Flow can even occur through a paper bridge (Fig. 4B). These results show how easy it is to add and remove media to or from circuits, and drive flow through them without using external pumps.

### Flow through tessellated hydrogel modules

**Fig. 5.**
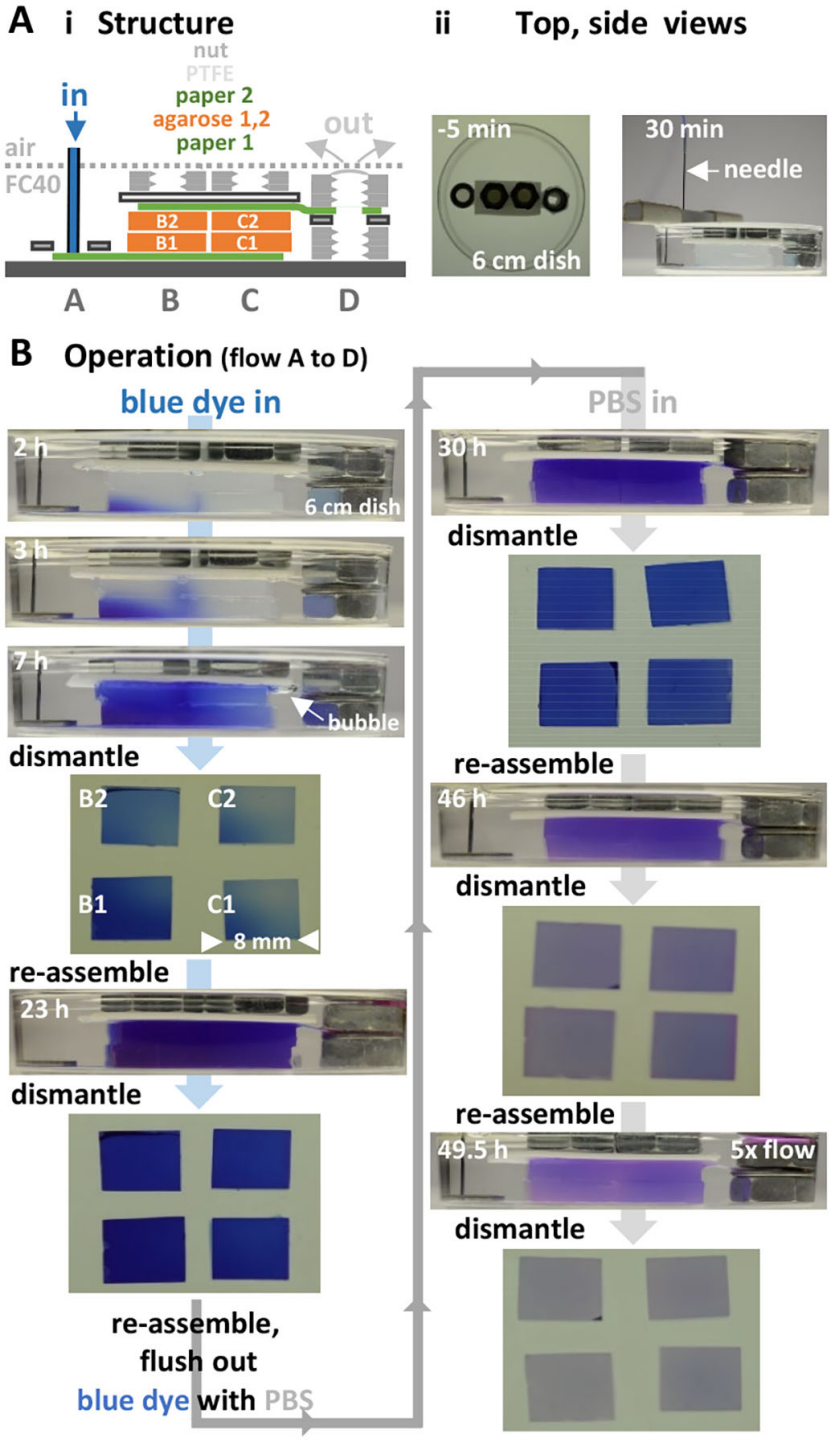
**Re-establishing flows after repeatedly assembling/disassembling stacks.** Times: operation time of pump. (A) Overview. (i) Structure. Stack A is for input. Stacks B and C each contain (bottom to top) filter paper 1, 3% agarose blocks B1+B2 or C1+C2 (each 8×8×3 mm) gelled in PBS, filter paper 2, a hydrophobic PTFE spacer, and an M6 nut as a weight. Stack D is for output. (ii) Top/side views before/after adding PBS+blue dye from a dispensing needle connected to a syringe pump. (B) Operation. Between 0 h and 7 h, the pump deposits PBS+blue dye onto the paper in stack A. The pump feeds the circuit at a constant rate; then flow is to the point of lowest pressure (the cap on stack D). The pump is now stopped, the circuit dismantled, isolated agarose blocks photographed, the circuit re-assembled, and the pump re-started. By 23 h, blocks are filled with dye. Again, the pump is stopped, dismantled blocks photographed, and the circuit rebuilt. Next, dye is flushed out of the system by depositing PBS without dye. By 30 h, blocks contain less dye. Two more cycles (the last using 5× flow input) flush out most dye.

We require that hydrogel-containing modules can be tessellated and aqueous continuity between them established; this proves to be easy. Thus, four blocks of agarose are assembled into abutting stacks (B,C in Fig. 5Ai) just by pushing them gently together. Next, phosphate-buffered saline (PBS)+dye is deposited steadily by a syringe pump on to a filter paper in stack A; dye flows through the filter paper under stacks B and C, upwards through blocks to an overlying filter paper that in turn takes it across a bridge to the cap on stack D (Fig. 5Ai,ii). The input is sufficient to replace the total agarose volume every ∼15 h; later, it is increased fivefold. Flow continues except during four cycles of circuit disassembly and re-assembly (Fig. 5B). Initially, PBS+blue dye fills the system, and then dye is flushed away by inputting pure PBS. Throughout, the cap on stack D repeatedly buds off as the system automatically self-empties at a rate balancing the input. After every disassembly, agarose blocks are imaged so dye passage through the system can be monitored, and the stack is re-assembled. As expected, flow takes the path of least resistance. Thus, after 3 h, some dye reaches filter paper 2 by passing between blocks B2 and C2, as lagging dye enters B1 (Fig. 5B). Even so, blocks generally fill successively (in order B1, B2, C1, C2), and dye is flushed out in the same order. Note the air bubble seen after 7 h; bubbles usually cause catastrophic failures in conventional microfluidic circuits (Pereiro et al., 2019), but here buoyancy soon expels them. These results show that aqueous connections are easily made and remade between tessellated hydrogel modules, and that the system can replenish media in stacked hydrogel modules (3 mm thick) in <1 day – and therefore support growth (in principle) of rapidly-dividing mammalian cells.

### Measuring and regulating flow

Darcy's law describes the flow rate, *Q*, of a fluid (dynamic viscosity *µ*) through a porous module of length *L*, permeability *k*, and cross-sectional area *A*; it depends on the pressure difference Δ*P* across that module, where Δ*P*=Q(µ*L*/*kA*). Here, µ*L*/*kA* is a resistance term, *R_hyd_*; then, Δ*P=QR_hyd_* (Kumar et al., 2019). As often noted, this is analogous to Ohm's law, where Δ*V*=*IR* (ΔV is potential drop, *I* current, and *R* electrical resistance; Kumar et al., 2019). Note also that application of Darcy's law requires use of appropriate boundary conditions. See, for example, Haber and Mauri (1983) and Lyaghfouri (1996) for some complexities associated with the use of different boundary conditions.

Flow patterns through tessellated modules in Fig. 5 are complex, and – as fluid walls inevitably morph during flow (Deroy et al., 2021b) – we need to define different boundary conditions from those applied hitherto and apply them to Darcy's law. In the meantime, we can draw some practical conclusions useful to biologists. Thus, to a broad approximation, flows are analogous to electrical ones through serial and parallel resistances, and – if there is a voltage/pressure difference across a circuit – some flow will occur through and around modules depending on their resistance/permeability. These flows may be the wanted ones immediately past cells growing in the middle of (high-resistance) modules, as well as wasteful ones through (low-resistance) gaps between modules or up the sides of stacked modules. However, cells should remain viable if they lie within diffusional reach of regions with high enough flows, irrespective of whether those flows are wanted or wasteful.

**Fig. 6.**
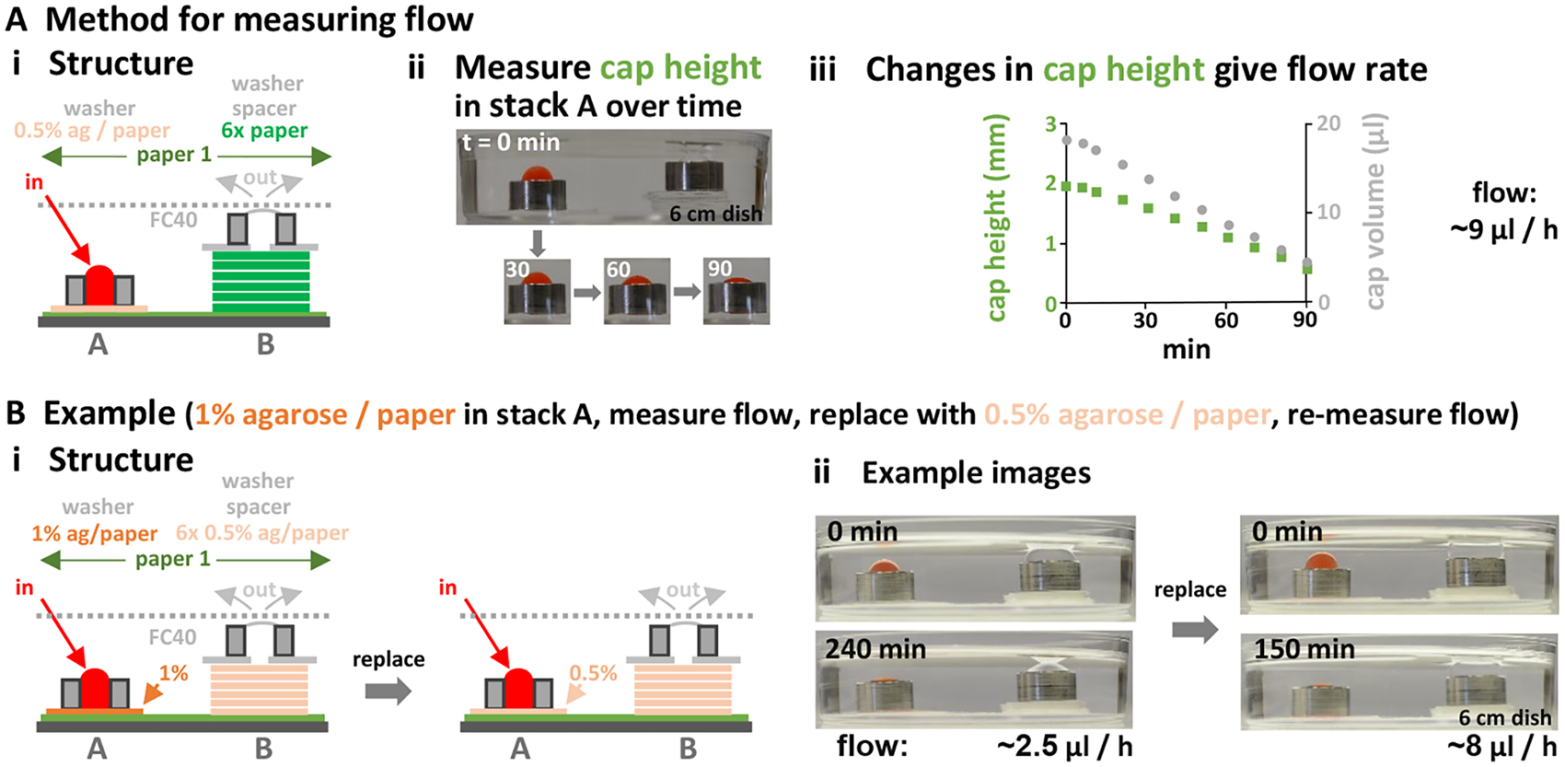
**Measuring and regulating flow (60 mm dishes; ‘ag’=agarose).** (A) Method. (i) Structure. Stacks A and B sit on filter-paper 1. Stack A contains a filter paper filled with 0.5% agarose to slow flow, plus a washer (height 5 mm, hole volume 73 µl) that acts as input reservoir. Stack B contains six filter papers capped by a washer. PBS+red dye is manually pipetted into stack A, which is imaged from the side. (ii) Images taken at different times (min). (iii) Changes in cap height, and derived changes in cap volume and flow rate. (B) Example: increasing flow by replacing a regulator with small pores (a filter paper filled with 1% agarose) by another with large pores (a paper filled with 0.5% agarose). (i) Structure. Stack A initially contains a regulator with 1% agarose. Stack B consists of six filter papers filled with 0.5% agarose. The rest of the circuit is initially filled with PBS, then red dye is pipetted into stack A to create a cap, images collected as the cap shrinks, and flow rates determined as in (A). (ii) Images taken before/after switching regulators; as expected, flow is fastest through the one with large pores.

Flow rates can be determined simply by measuring how quickly input caps shrink. For example, using a two-stack circuit (Fig. 6Ai), images are collected from the side (Fig. 6Aii), input cap height measured, cap volume determined (assuming a spherical cap), and the rate of reduction in cap volume (and so flow rate) derived (Fig. 6Aiii; both rates decline over time as the system equilibrates). A second and quicker way of assessing flow is to fill the output reservoir to the brim so the cap just becomes visible from the side (at t=0), and – once the cap has filled (at t=x) – remove volume back down to the level at t=0 (this should include any medium that has budded off to float on the surface); then the flow rate is the recovered volume per unit time.

Flows through circuits can be varied by introducing regulators. For example, replacing a high-resistance filter-paper filled with 1% agarose at the base of an input stack by a low-resistance one filled with 0.5% agarose (Fig. 6Bi, left) speeds flow through a simple circuit ∼three fold (Fig. 6Bii). Similarly, replacing a Millipore filter with 0.1 µm pores by another with 0.2 µm pores speeds flow ∼10-fold (Fig. S4). Note that when medium contains fetal bovine serum (FBS), aggregates in serum progressively clog sub-micron pores; then, clogged regulators can be pierced by a syringe needle or replaced by fresh ones (as in Fig. 6B). Several inputs into – and outputs from – circuits can also be added if wanted (Fig. S5). These results show that flow rates into, through, and out of circuits can be controlled over a wide range and at various points.

### Clonal growth of HEKs in modules containing Geltrex

In principle, modules can be made of any appropriate biomatrix (Caliari and Burdick, 2016) that can be picked up and stacked. Materials too fragile to be handled can also be cast in stronger scaffolds; for example, we have filled stainless-steel and paper washers with agarose, collagen, Matrigel, Geltrex, alginate, and cellulose (both nano-fibrillar and nano-crystalline). Silk is an attractive scaffold as it is composed mainly of fibroin that is strong, biofriendly and biodegradable. When single human embryonic kidney (HEK) 293 cells are cast in Geltrex to fill cavities in silk meshes (10×10 mm, ∼211 µm thick), clones grow and develop in the hydrogel like their conventionally grown counterparts (Fig. S6A). Similarly, HEKs clone normally in nylon meshes (10×10 mm, 340 µm thick), either unstacked and immersed in medium, or in stacks of five (Fig. S6B). This shows that cells grow normally in hydrogels held in various scaffolds, even when 5 mm from stack sides and 850 µm from top or bottom.

**Fig. 7.**
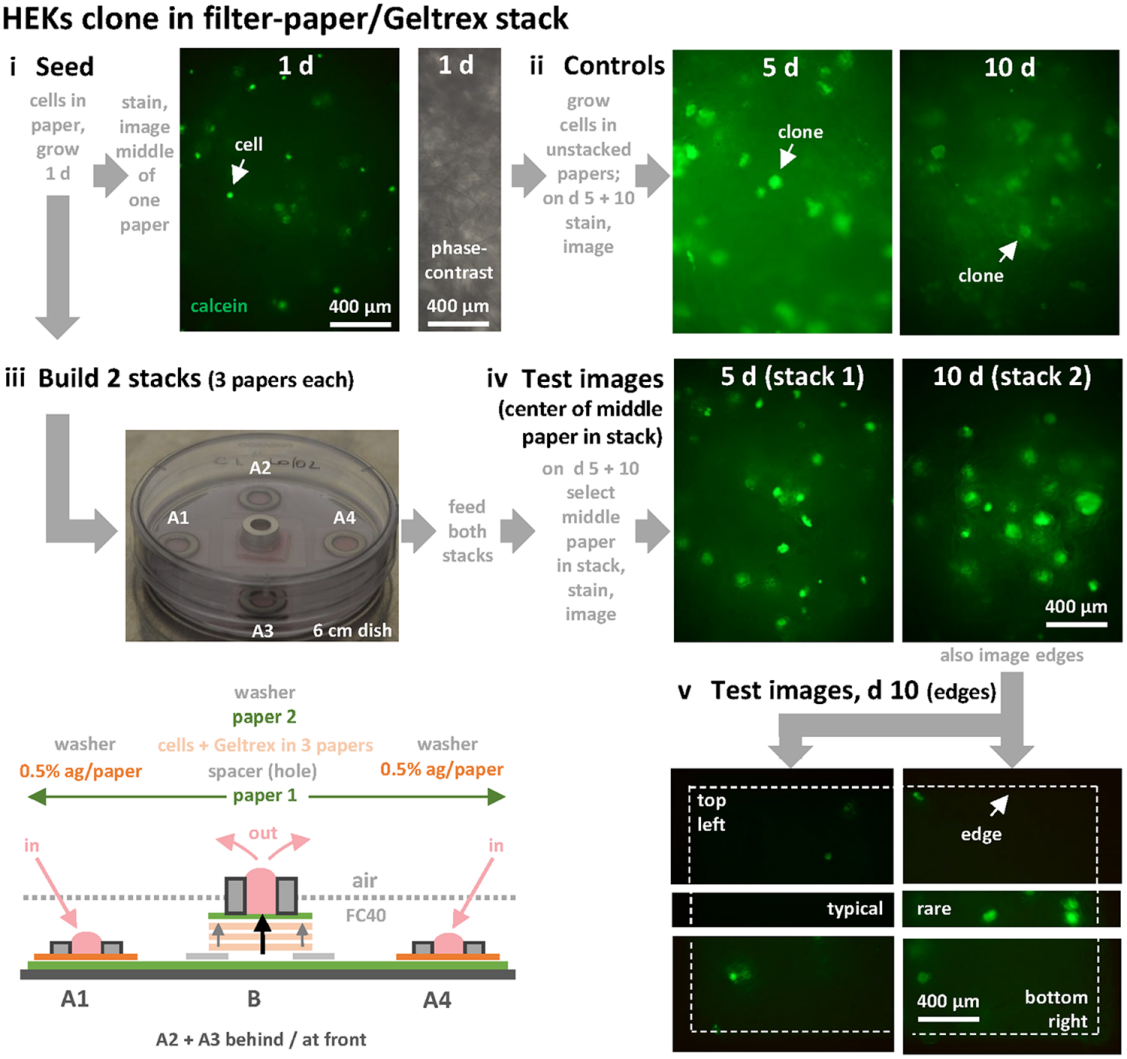
**Clonal growth in filter-paper stacks demonstrated using calcein AM, a non-fluorescent ester converted by living cells to green-fluorescent calcein.** Only a few cells/colonies lie in focal planes, and images collected on different days are not of the same field (papers are discarded after staining). (i) Seeding HEKs in filter-paper squares. Squares (10×10 mm; thickness ∼340 µm) are dipped into ice-cold medium+10% Geltrex containing HEKs (10^5^/ml), so the mixture wicks into papers, and squares immediately dropped into medium (37°C) to set the gel. Cells in unstacked squares immersed in medium are grown (1 d), stained, and fluorescent (left; arrow marks one cell) and phase-contrast images (right; cells cannot be detected against the background due to the variable paper density) of centers of a square collected. (ii) As controls, cells in unstacked squares immersed in medium are grown (fed by diffusion) for 5 and 10 days, and cells stained and imaged; fluorescent colonies enlarge over time (arrows mark colonies). (iii) Two sets of three squares are stacked on day 1. Image: central stack of three squares surrounded by four inputs (A1–A4), all sitting on an octagonal paper base. Cartoon: medium is pipetted into inputs A1–A4 so hydrostatic pressure drives it down through regulators to the base, laterally to stack B, up through the hole in a relatively impermeable spacer and past cells in a thin hydrogel in the three squares, to the cap. Upward flow in stack B is higher in the middle (black arrow) compared to the sides (grey arrows). (iv) For the experiment, medium is pipetted daily into A1–A4, squares removed from stacks and stained on day 5 (stack 1) and day 10 (stack 2), and imaged. Large fluorescent colonies develop much as in controls in the middle of central papers. (v) Images of the edges of the central paper in the second stack on day 10. No live cells/colonies are generally detected (‘typical’), although a few single cells are seen at corners; ‘rare’ illustrates the largest fluorescent foci seen anywhere within 1.2 mm of an edge.

Paper is especially attractive as a scaffold when validating our platform as it is so widely available as sheets with thicknesses and pore sizes in our desired range (Fu and Wentland, 2022). Therefore, we next cloned HEKs in stacks of Whatman filter papers with pores larger than cells (i.e. number 113; thickness ∼340 µm – this paper retains particles down to 30 µm according to the manufacturer's data, contrasted with number 1, which is ∼180 µm thick and retains particles down to 11 µm). The circuit contains four input stacks with agarose:paper regulators (A1–A4) that feed the central stack B (Fig. 7; see Fig. S7 for visualization of dye flows through this circuit, and for an improved circuit with less wasteful flow). Paper squares (10×10 mm) are picked up with tweezers, dipped briefly into ice-cold Geltrex plus HEKs. This hydrogel is liquid at this temperature (but gels at 37°C), so the liquid mixture wicks immediately throughout. Next, squares are quickly dropped into medium at 37°C to set the gel. Here, the Geltrex concentration is too low to yield a free-standing gel but sufficient to create a very thin gel that we assume is stable in pores in the paper. After growth for 1, 5, or 10 days in unstacked papers immersed in medium in a dish, live cells are imaged within squares using calcein AM, a cell-permeant and non-fluorescent ester converted by living cells (but not dead ones) to green-fluorescent calcein. Initially, single cells are spread throughout the paper; subsequently, clones grow throughout these control papers as all cells lie <200 µm from the exterior (Fig. 7i,ii). Two circuits are also constructed on day 1, each with a central stack of three cell-containing papers sitting on a water-impermeable spacer with a square hole (6×6 mm). This hole should ensure a high flow up through the middle, and low flow near edges (Fig. 7iii, grey and black upward arrows). This flow-poor zone serves as an internal control for the central flow-rich one. After 5 days, the paper square in the middle of the first stack is removed and stained, and its middle imaged; this process is repeated with the second stack on day 10. Importantly, we remove lids from dishes in a sterile biosafety cabinet only during stack assembly and subsequent manual feeding by pipetting; after transfer to a CO_2_ incubator, refreshing flows up stacks plus self-emptying then occur automatically with lids in place. Colonies again develop in the centers of both stacks much like those in unstacked control squares (compare Fig. 7ii with Fig. 7iv). However, in contrast to normal growth in the center of the stack, no colonies are generally seen at edges in flow-poor zones (Fig. 7v, ‘typical’ panel), and – if they are seen – they are always small (Fig. 7v, corner and ‘rare’ panels). Cells also clone normally in a taller stack (height ∼1.7 mm; Fig. S8i-iv). If a stack is not fed for 10 days, cells also fail to grow, as there is too little medium in an unfed stack to support growth (Fig. S8v). These results validate the workflow outline in Fig. 1, and confirm the expectation that refreshing flows are required to sustain growth in stacked modules with dimensions greater than the diffusion limit.

**Fig. 8.**
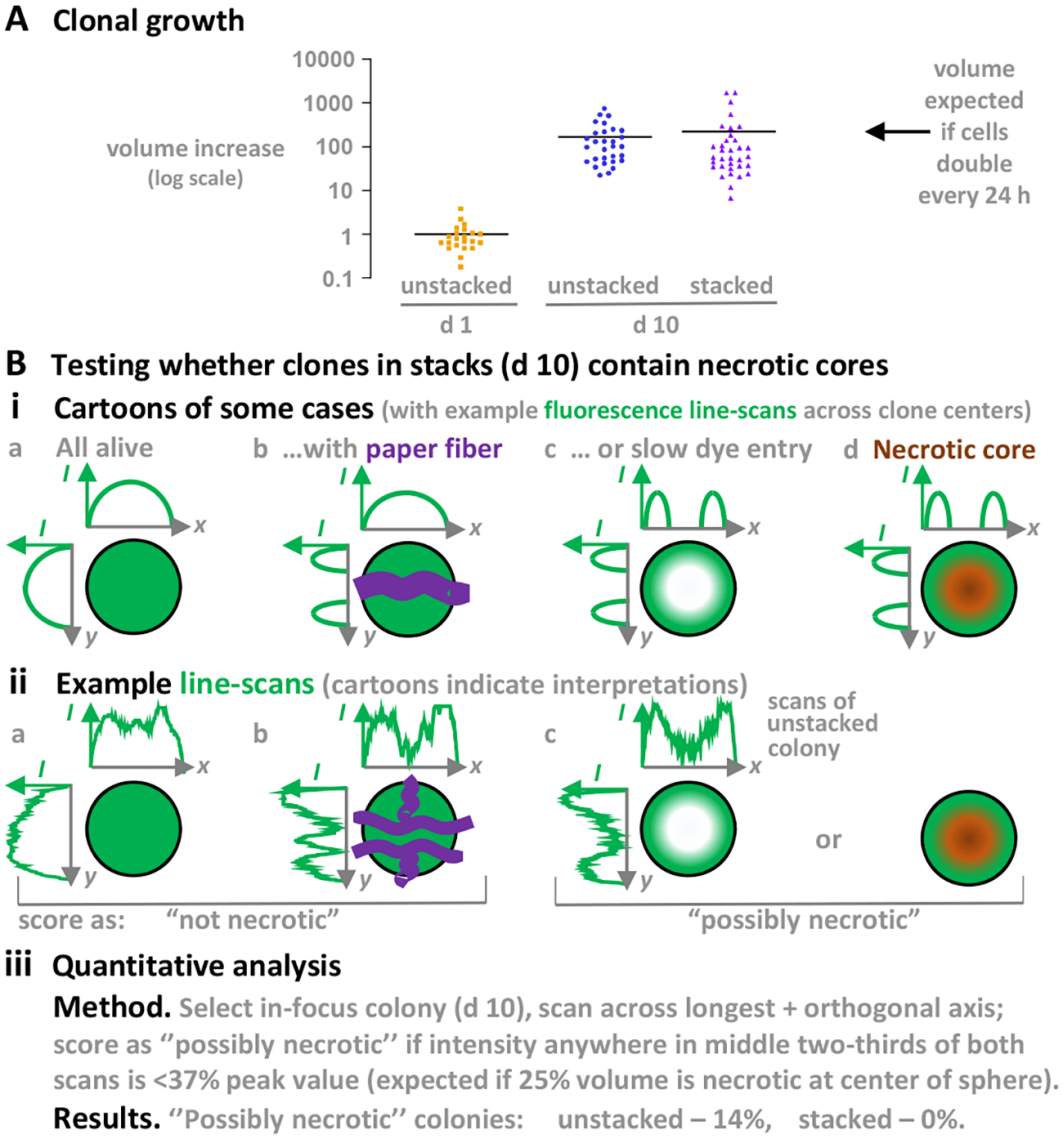
**Comparing the growth and wellbeing of colonies.** Data are from experiments illustrated in Fig. 7 (panels i, ii and iv) and S8 (panels i, ii and iv), where filter-paper squares containing cells (day 1) or colonies (day 10) are stained with calcein AM, fluorescence images of 10 different fields in the middle of each paper collected, in-focus colonies selected (usually ∼2/field), and fluorescence areas and line scans across colonies analyzed. We assume that a fluorescent focus seen on day 1 contains one cell (some cells may have divided since seeding the previous day), and that colonies on day 10 completely fill a spherical colony (Fig. 7 and Fig. S8 shows colonies are often non-spherical). (A) Clonal growth. Cell and clone volumes are calculated from areas in images. Cell volumes increase in both stacked and unstacked papers at rates close to those expected if cells double every 24 h. We conclude that growth in stacks (height ∼1020 and ∼1700 µm) is comparable to that in unstacked ones (thickness ∼340 µm). (B) Testing whether clones in stacks contain necrotic cores. Single living and dead cells are often distinguished using the LIVE/DEAD Viability/Cytotoxicity Kit (ThermoFisher Scientific). The calcein AM used in these experiments is from this kit, and is used to detect living cells. Unfortunately, the red-fluorescent ethidium homodimer-1 (provided in the kit to detect dead cells) proved unusable as it binds to paper (giving high backgrounds). (i) Cartoons illustrating some challenges associated with determining the degree of necrosis. (a) If a spherical colony is completely filled with live cells, staining with calcein AM should yield orthogonal (*x*,*y*) fluorescence line-scans giving the intensity (*I*) profiles indicated. (b) If such a colony grows around a paper fiber (purple), a ‘valley’ is found in one profile. (c) Live-cell staining involves incubation for 1–2 h, and the dye may not have time to diffuse into the middle of the colony. Consequently, both profiles might contain central valleys despite all cells being alive. (d) Staining a necrotic colony also gives two profiles with central valleys (as c). We will argue that colonies with a central necrotic core should have central valleys in both profiles. (ii) Three example line-scan pairs (intensities and widths are normalized, so peak intensity and scan length=100 arbitrary units), plus interpretative cartoons. (a) Neither profile has a ‘valley’ – consistent with no necrosis. (b) The *x* profile has a ‘valley’ and the *y* one a central peak – consistent with no necrosis and fibers as indicated. (c) Both profiles have valleys, consistent with non-necrotic and/or necrotic colonies. This profile is from a freely-floating (unstacked) paper, as no such profiles were seen in stacked papers. (iii) Quantitative analysis. We assume a colony is ‘possibly necrotic’ as indicated. We conclude that colonies in stacks are at least as free from necrosis as those growing in unstacked papers.

Finally, we would like to quantify cloning efficiencies, growth rates, and whether or not colonies contain necrotic cores; however, each one of these proves challenging with any approach used for organoid culture, including ours – largely because so many assumptions are involved. These challenges include where to draw thresholds when imaging living objects many hundreds of microns thick, and accounting for the degree of penetrance into colonies of the dyes used to monitor necrosis. In the experiments described in Fig. 7 and Fig. S8, there are additional complexities introduced by, for example, local variations in paper density (that scatter light to different degrees and give significant fluorescent halos around colonies), and the unusably high backgrounds given by the most widely used dye used to monitor necrosis (Red-fluorescent Ethidium Homodimer-1 binds to Whatman filter papers, as well as necrotic cells) – which prevented us from using it. Against this background and after making many assumptions, in Fig. 8 we describe attempts to determine cloning efficiencies, growth rates, and degree of necrosis. We conclude that cells seeded in paper appear to clone and grow at rates found conventionally to yield colonies with non-necrotic cores, and that these colonies are of equal size whether or not they are grown in stacks.

## DISCUSSION

Our aim is to establish a platform for culturing micro-organoids in standard Petri dishes. Recognizing that organs develop *in vivo* from layers of different cell types under critical length constraints, each cell type is grown in a different module, and then modules are stacked appropriately (Fig. 1). Evaporation of small volumes is limited by confining the aqueous phase behind fluid walls made of an immiscible fluorocarbon (FC40), and interfacial forces pin modules to dishes and prevent medium from leaking out (Fig. 2). Additionally, aqueous connections between modules are created automatically on contact, and refreshing flows through and around modules can be driven without using external pumps by exploiting differences in hydrostatic pressure (Figs 3 and 4). Such flows can be from several inputs (Fig. S5A) through modules tessellated vertically or horizontally (Fig. 5) to several outputs (Fig. S5B). Flow rates can also be regulated over a wide range (e.g. Figs 4 and 6) through modules with different permeabilities (e.g. paper, agarose, Geltrex; Figs 5–8). In proof-of-concept experiments, we show HEKs clone as expected in hydrogel stacks up to 1.7 mm high and 10 cm wide (Fig. 7; Fig. S6B, S8i-iv).

This platform has many advantages (Table S1). Probably the main one is that it uses an open and forgiving form of microfluidics accessible to bioscientists. For example, circuits can be fabricated in minutes using equipment available in most biolabs, and liquids can be added to them (or withdrawn from them) using standard pipettes. Moreover, cells are grown in standard CO_2_ incubators as FC40 is freely permeable to O_2_ and CO_2_. Note that FC40 also carries tenfold more O_2_ than water without binding the gas (Sarkar et al., 2014; Krafft and Riess, 2015), so this approach should prove advantageous when growing O_2_-demanding brain organoids (Lancaster et al., 2013; Kelley and Pasca, 2022). Just as cell biologists check cell welfare by observing the acidity and sterility of media in their dishes, we do so similarly by observing caps on stacks. And just as they monitor development at the center of organs by sectioning and imaging on a standard microscope, we do so by dismantling stacks and imaging central modules – and we can even rebuild stacks to continue experiments and/or swap in or out new modules (Figs 5, 6B). However, our approach still needs development (see Fig. S7B for an improved circuit design that has larger input reservoirs and slower flow rates, and reduces the number of times that media needs to be refreshed), and has disadvantages. Probably the main drawback is that many hydrogels in wide use are not robust enough to be stacked and unstacked in the ways we describe, so we incorporate them into stronger supporting scaffolds (fortunately, strong composite hydrogels are being developed; Sánchez-Cid et al., 2022). Second, our modules currently lack a vascular system (Lee et al., 2018; Wimmer et al., 2019; Zhang et al., 2021), but there is every chance that introducing appropriate endothelial cells will lead to vasculogenesis, as pre-existing flows enhance this (Homan et al., 2019). Therefore, we hope the advantages and simplicity of this modular Lego-like platform will enable more bioscientists to miniaturize organoid culture.

## MATERIALS AND METHODS

### General materials, equipment

All materials are from Merck (Darmstadt, Germany) unless stated otherwise. FC40 was from Acota (Shropshire, UK), stainless-steel nuts and washers from a local hardware store (Robert Dyas, Oxford, UK) or Accu Ltd (Huddersfield, UK), Teflon washers from Washersdirect (Birmingham, UK), tube magnets from Magnet Expert Ltd (Tuxford, UK), monofilament nylon mesh (400 µm opening, thickness 340 µm) from Cadisch Precision Meshes Ltd (Herts, UK), and natural silk mesh CQ15 (15 mesh/cm, 457 µm opening, open area 47%, thickness 211 µm) from Bonfilt (Qingdio, China). Water-soluble dyes (e.g. Allura Red AC, resazurin; concentrated stock solutions are filtered through 0.2 µm Millipore filters to remove particulates and provide sterility) are used where indicated.

Liquids are sometimes delivered through blunt dispensing needles (25 G, OD 500 µm; Adhesive Dispensing, Milton Keynes, UK) connected via a PTFE tube (26 G; Adtech, Stroud, UK) to syringe pumps (1 or 5 ml syringes; Harvard PHD Ultra I/W; Harvard Apparatus, Holliston, MA, USA). When high flow rates are used, needles sometimes have PTFE collars at their tips to prevent flow of inputted aqueous phase upwards (this is not usually necessary). Needles are held in 3D-printed rectangular rods containing holes, and rods are surrounded by double-sided sticky tape so they can stick to the top sides of dishes. Before starting pumps, needles are lowered to contact prewetted filter papers in input stacks; after starting pumps, inputs are delivered to the filter paper, and then differences in hydrostatic pressure are the main drivers of flow through circuits).

Images of dishes are taken using a digital SLR camera (Nikon D610). Bright-field, phase-contrast, and fluorescent images of modules are collected using a digital SLR camera (Nikon D7100) connected to an epi-fluorescent microscope (Olympus IX53; 4X, 10X; FITC filter) equipped with a CoolLED pe-100 combiner and 470 nm light source (Andover, UK).

### Cells

Adherent HEK 293 are grown routinely in DMEM+10% FBS (Gibco, Life Sciences)+1% penicillin plus streptomycin (P/S, Gibco) – which is referred to as ‘medium’ throughout, and sub-cultured using trypsin (TrypLE, Gibco #12563011, Gaithersburg, MD, USA). They were obtained from the departmental cell bank (which tests for mycoplasma prior to freezing), but they were not tested after thawing or authenticated in any way. Medium is supplemented with 10 mM HEPES when cells are grown in stacks. Geltrex was from ThermoFisher Scientific (Loughborough, UK).

### General principles: building and operating circuits

Here, we describe general principles (the operation of individual circuits is described below). (1) Polystyrene dishes (60 mm; Corning #430589, 60 mm×15 mm style, suspension culture dishes, non-treated for tissue-culture) are used initially unless stated otherwise, as we thought their use would minimize aqueous flows around components sitting on a dish. However, these dishes became unavailable at one stage during the COVID-19 pandemic, and we found dishes treated for tissue-culture gave similar outcomes (Figs S7B, S8). (2) The bottom component in a stack is always hydrophilic; it is usually a Whatman filter paper pre-wetted with PBS or medium to ensure the aqueous phase in the stack is held by interfacial forces (acting in the component) to a dish when FC40 is overlaid. (3) Stacks are often built by adding one hydrophilic layer, wetting it with medium or PBS, adding FC40 to prevent seepage, then adding the next hydrophilic layer, etc. (as wetting with FC40 can prevent subsequent adherence of an aqueous phase; e.g. Fig. 2C). (4) PTFE spacers (washers or rectangles cut from a sheet with thickness 1 mm; Direct Plastics, Sheffield, UK) are used initially between two hydrophilic layers to minimize flow around the outside of the hydrophilic components. Subsequently, these are replaced by hydrophobic spacers made by cutting ‘Rite in the Rain’ paper (JL Darling LLC, WA, USA; purchasable from craft stores; ∼110 µm thick) to the desired 2D shape, and pre-soaking it in FC40 to exclude water completely (FC40 wicks rapidly throughout); then, the flow rate through this paper (determined as in Fig. 6A) is essentially zero. Alternatively, this paper can be pre-wetted with medium, to create the relatively impermeable barrier used in Fig. 7, Fig. S7, and S8. This paper wetted with medium can also be used as a flow-regulator (Fig. S7B; flow rate determined as in Fig. 6A is ∼4.3 µl/h). (5) A hydrophobic layer with a central hole often overhangs a lower hydrophilic module to maximize upward flow through the module and minimize it around the outside (as at the base of the central stack in Fig. 7iii). (6) While modules are stacked and aligned manually (the ‘Cheerios’ effect aids alignment), the system is forgiving of imperfect alignment (e.g. see Fig. 5B). (7) The topmost component in a stack is often a weight added to prevent underlying components from detaching from the bottom of a dish when adding dense FC40. (8) Peepholes are often included to provide visual access to stack interiors; this is why we use washers/nuts with central holes as weights, and cut holes in filter papers and ‘Rite in the Rain’ spacers (often using a standard office hole-punch). (9) Fluorescent microparticles (10 µm diameter; carboxylate-modified, rhodamine-marked) can be used as surrogate cells when testing wicking through modules and hydrogel gelling rates within them. (10) FC40 is sterilized by filtration, and steel/PTFE washers/nuts, filter papers, ‘Rite in the Rain’ paper, and nylon/silk meshes by autoclaving (when paper and mesh squares can be sandwiched between strips of aluminium foil to minimize bending). (11) When manually pipetting medium into reservoirs (e.g. in Fig. 7), the medium can rise up the outside of a vertical tip to float on the FC40. Therefore, we hold the pipette tip as far from the vertical as possible, lower it until it touches the filter-paper base, eject medium slowly onto the base, and remove the tip by raising it close to the hydrophilic reservoir wall. In addition, the outside of a tip can be pre-wetted with FC40 to minimize such adherence to the outside: an empty tip (loaded on a pipet) is dipped into FC40, air ejected, the tip filled with fresh medium, and medium ejected as described above. (12) A circuit can support flows through modules (without flows around them) only up to a certain limit; much as high inputs cause rivers to overflow their banks, inputs above this limit cause wasteful flows over surfaces and around modules in our platform (Fig. 5, Fig. S3, S5, S7Aii; see Fig. S7B for more detail). Such limits are best determined by progressively increasing inputs using an external pump. When these limits are exceeded, medium can flow over regulators and filter-paper bases to puddle on the surface of the base and at the edge of the bottom module in a stack before ballooning up from the base or flowing wastefully up around modules (e.g. Fig. S3B last image, Fig. S5B 9 h image); over time, these flows decline as the system equilibrates (e.g. Fig. S7A). Naturally, we want to maximize volumes passing through modules, and minimize wasteful flows around them – all while ensuring flows are as uniform as possible (Fig. S7B illustrates ways towards this). Therefore, we visually check (immediately after inputting) how quickly input caps shrink and whether puddles form (especially on the filter-paper base at the bottom of stacks), and eject small inputs slowly over a long period (instead of one large one quickly that is obviously more convenient). We also use flow regulators to minimize the size of the surge immediately after inputting (as in Fig. 7; Figs S7 and S8).

### Building and operating individual circuits

For Fig. 4A (where dye flows from stack A to B through a filter paper on the bottom of a dish), a rectangular filter paper (30×10 mm; Whatman number 1) is placed on a 60 mm dish, coaxial stacks of two and three stainless-steel washers (OD 10 mm, ID 5 mm, thickness 1 mm) placed at each end (Fig. 4Aii), the filter paper wetted (50 µl medium), each stack filled with 25 µl medium, and 15 ml FC40 overlaid (Fig. 4Aiii, −1 min). Medium plus blue dye (50 µl; 50 µg/ml resazurin) is pipetted through FC40 on to the filter paper in stack A, followed by 50 µl dye after 5 min (Fig. 4A, 5 min); then, 25 µl aliquots of dye are added every 5 min.

For Fig. 4B (where dye flows through a filter-paper bridge), two stacks (A and B) of three stainless-steel washers (OD 10 mm, ID 5 mm, thickness 1 mm) are placed with axial centers 30 mm apart on the bottom of a 60 mm dish; stack A is filled with 75 µl medium+blue dye (as for Fig. 4A), and stack B with 75 µl medium (Fig. 4Bii). A rectangular filter paper (30×10 mm; Whatman number 1) pre-wetted with medium is then laid over stacks; one washer is placed as a weight to complete stack A, and three washers to complete stack B. FC40 (20 ml) is overlaid to cover both stacks, and medium plus blue dye (25 µl aliquots) pipetted every 5 min onto the filter paper in stack A (Fig. 4Biii).

For Fig. 5 (where stacks B and C each contain two agarose blocks): (1) A rectangular filter paper (28×8 mm; Whatman number 1) is placed on a 60 mm dish, wetted with 25 µl PBS, and most air bubbles removed by pressing on the paper. This paper (filter paper 1) constitutes the bottom layer of stacks A–C. (2) Stack A is completed by placing the center of a stainless-steel washer (OD 10 mm, ID 5 mm, thickness 1 mm) on the midline of filter paper 1 at the left-hand end. (3) The second layer of stacks B and C consists of blocks B1 and C1 [each 8×8×3 mm and made of gelled 3% agarose (low-melting point, Sigma-Aldrich type VII) in PBS]. Blocks are placed wet with their centers on the midline of filter paper 1. The right-hand edge of C1 extends ∼2 mm over the right-hand edge of filter paper 1, and the right-hand edge of B1 abuts the left-hand of edge of C1 to provide aqueous continuity between the two. This placement ensures there is space between the washer in stack A and block B1, and that each block overhangs the sides and left-hand end of filter paper 1 (to minimize flow up/around outsides of agarose blocks). (4) The third layer of stacks B and C consist of blocks B2 and C2 (B2 on B1, and C2 on C1). B1 and B2 are gently pushed sideways on to C1 and C2 to ensure lateral aqueous continuity. Additional layers will be added later to stacks B and C. (5) Stack D is built several millimeters to the right of half-built stack C. A stainless-steel M6 nut (width across flats 10 mm; thickness 5.2 mm) is placed on the dish, and then a stainless-steel washer (OD 10 mm, ID 5 mm, thickness 1 mm) co-axially on top of the nut. (6) Filter paper 2 (identical to filter paper 1 except for a hole) is pre-wetted with PBS, and added on top of B2, C2, and the washer in stack D. The left-hand edge of filter paper 2 is positioned ∼2 mm to the right of the left-hand edge of block B2. Filter paper 2 has a rectangular peephole (4×3 mm) with its right-hand edge 3 mm away from the right-hand end, so the hole lies over holes in the washer and nut below. (7) PBS (100 µl) is pipetted from above directly through co-axial holes in growing stack D on to the bottom of the dish (where it is held by interfacial forces). (8) FC40 (4 ml) is added to the dish to cover the washer in stack A (to minimize seepage of PBS from nascent stack D). Interfacial tension plus the effects of gravity hold the circuit on the bottom of the dish. (9) A second M6 nut is added to complete stack D. (10) A solid PTFE rectangle (25×13×1 mm) is placed over filter paper 2 above blocks B2 and C2. It provides an overhanging roof that acts as a spacer between the paper and two M6 nuts that will be added later. This rectangle is gently pressed down on to nascent stacks B and C to ensure there is vertical aqueous continuity between underlying filter papers and agarose blocks. Stacks B and C are completed by adding an M6 nut to each stack on top of the PTFE rectangle. (11) FC40 (5 ml) is added to fill the dish up to a level about half-way up block B2, 100 µl PBS is pipetted into the PBS already in stack D, and the dish filled with FC40. Pressures in the system equilibrate over ∼15 min. As unknown amounts of PBS are added with pre-wetted filter papers and agarose blocks during building, sometimes a cap of PBS appears at the top of stack D, and sometimes this shrinks/expands during equilibration depending on volumes present. It is convenient to add/remove PBS to this cap by pipetting through FC40 so the top of the cap can just be seen from the side above the top of the top-most nut in stack D; then, when PBS +/− dye is pumped into stack A, an increase in cap size allows easy monitoring of successful transfer of aqueous phase through the system. (12) Fig. 5Aii gives a top view of the filled dish at this stage. Note that the plastic dish is hydrophobic, PTFE is fluorophilic and hydrophobic, and stainless steel is hydrophilic. The arrangement exploits these properties to minimize seepage of PBS under, over, and around filter papers and agarose blocks, and to contain PBS in input and output reservoirs. (13) The tip of a stainless-steel dispensing needle (without a PTFE sleeve) is lowered down through the hole in the washer in stack A until it touches the wetted filter paper 1. The needle is also filled with PBS plus blue dye (0.4 mg/ml resazurin) and connected to a syringe pump (1 ml syringe). Once the pump starts, PBS plus blue dye is delivered (50 µl/h) to filter paper 1 in stack A. Thereafter, PBS+dye automatically flows to the cap on stack D (from which it is removed manually with a pipette during the day, or the cap buds off and floats to the surface overnight). Fig. 5Aii also illustrates a side-view of the set-up 30 min after starting the pump; close inspection reveals that some blue dye has already passed along filter paper 1 and is entering B1. (14) The circuit is dismantled as follows: the pump is stopped, the dispensing needle raised, stack D emptied of PBS (+ any dye) by manual pipetting, the dish emptied of FC40, stacks dismantled (top down), the four agarose blocks and two filter papers washed free of surface PBS by passing through FC40, FC40 drained from surfaces, and blocks placed on a 60 mm dish and photographed. (15) The circuit is reassembled as follows: the four agarose blocks plus two filter papers are rinsed briefly in PBS, reassembled into stacks, and PBS±dye pumped in. Subsequently, stacks are disassembled, imaged, and reassembled as before.

For Fig. 6A, a flow regulator is made by pipetting 50 µl molten agarose (0.5% Ultrapure, Invitrogen, in PBS) onto a filter paper (15×15 mm, Whatman number 1) in a bacteriological dish (90 mm) on a hotplate at 65°C; after pipetting, the dish is immediately transferred to ice-cold agarose gels. Subsequently, the filter is sterilized by incubation overnight in 70% ethanol, ethanol removed by transfer through three sets of sterile PBS (5 ml), and stored sterile at 4°C until use (although sterility is not required here). The circuit is built by placing a filter-paper (Whatman number 1, 40×20 mm) on a 60 mm dish and wetting the paper with PBS. Next, input stack A is built by adding successively the flow regulator and a stainless-steel washer (OD 8 mm, ID 4.3 mm, height 5 mm), and output stack B by placing six filter-paper rectangles (Whatman number 113; 15×15 mm, thickness ∼380 µm), followed by a washer (OD 8 mm, ID 4.3 mm, height 5 mm). This type of filter paper is chosen because it retains particles of ∼30 µm (most mammalian cells are smaller than this, and so should wick throughout it). Washers in both stacks are filled with PBS (ensuring stack B becomes fully wetted), and FC40 added to cover the top of the washer in stack B. Next, red dye (0.4 mg/ml in PBS is pipetted into the washer in stack A, and the volume adjusted to leave a spherical cap visible from the side. Over the next 90 min, images are collected from the side (the optical axis of the camera is level with the top of the washer in stack A). Flow rates are determined at different times during the experiment by measuring heights of the middle of the spherical cap above the top of the washer in stack A in images (using ImageJ; Rasband, 1997–2018), and then calculating changing cap volumes seen in successive images.

For Fig. 6B, flow regulators containing 1% and 0.5% agarose in stack A are made as described for Fig. 6A except that 45 µl molten agarose is pipetted on to a filter-paper squares (15×15 mm), and 20 µl on to others of 10×10 mm. The resulting squares are not sterilized. Filter papers containing 1% agarose for stack B are made like the 10×10 ones in stack A. The circuit is made and operated as follows. A filter paper (40×20 mm, Whatman number 1) is placed in a 60 mm dish and wetted with 120 µl PBS. Input stack A is built by adding successively a regulator containing 1% agarose (15×15 mm) and a washer (OD 8 mm, ID 4.3 mm, height 5 mm). Stack B is built using five filter papers containing 1% agarose (10×10 mm), an impermeable spacer (12×12 mm with a central peephole with diameter 5 mm; ‘Rite in the Rain’ paper pre-soaked in FC40), and a washer (O.D. 8 mm, I.D. 4.3 mm, height 5 mm). Caps in both washers are filled with PBS, and FC40 added to cover the top of the washer in stack B. After 30 min equilibration, red dye (0.4 mg/ml Allura Red in PBS) is manually pipetted into the washer in stack A, and the volume adjusted to leave a spherical cap visible from the side. Over the next 240 min, images are collected from the side and flow rates determined. Next, FC40 is removed, stack A dismantled and rebuilt by replacing the regulator with 1% agarose with another of the same size that contains 0.5% agarose, the cap in stack A refilled with red dye, images collected for the next 150 min, and flow rates determined. Here, the fastest rate replenishes aqueous contents of the six modules every ∼3 days.

For Fig. 7, filter papers (Whatman number 113; particle retention 30 µm; thickness of this crepe paper varies between ∼240–440 µm) are cut into squares (10×10 mm). Squares are picked up with tweezers, dipped briefly into an ice-cold mixture of HEKs (1 volume at 10^6^/ml)+Geltrex (1 volume)+medium with 10 mM HEPES (8 volumes) so the mixture wicks throughout, dropped immediately into 8 ml DMEM+10 mM HEPES (37°C) to set the gel, and submerged papers incubated overnight (37°C). This Geltrex concentration is too low to yield free-standing gels but forms a dilute gel in paper pores. Next day, live cells are imaged using one half of the LIVE/DEAD Viability/Cytotoxicity Kit for mammalian cells (ThermoFisher Scientific). The unused half exploits binding of Red-fluorescent Ethidium homodimer-1 to indicate loss of plasma membrane integrity, but the homodimer binds to paper giving a high background. The used half exploits calcein AM, a cell-permeant and non-fluorescent ester converted by living cells to green-fluorescent calcein. Thus, papers are rinsed briefly in PBS, incubated at room temperature in 1 µM calcein AM, and phase-contrast and fluorescence images (1 s exposure, 85% contrast) collected within 1–2 h of adding calcein. Controls are cells grown in submerged and unstacked papers (three per dish in 8 ml medium) for 5 or 10 days (they are fed by diffusion). Stacks are constructed (day 1) on filter-paper 1 (an octagon of Whatman number 1; in Fig. 7ii, four of the eight edges are 20 mm, and the distance between the centers of two of these opposite edges is 47 mm); 500 µl is deposited in the center of a 60 mm dish, and the octagon overlaid. Input stacks A1-A4 consist of a flow-regulator capped by a stainless-steel washer (OD 10 mm, ID 5 mm, thickness 1 mm). Regulators are made by pipetting aliquots (30 µl) of molten agarose (30 µl of 0.5% in PBS; Ultrapure, Invitrogen) on to a filter paper (Whatman number 1; 12×12 mm) sitting on a bacteriological Petri dish on a hot-block at 65°C, transferring the dish to ice for 10 min to set the agarose, storing in 70% ethanol (4°C) until use, rinsing the now-sterile papers twice in PBS, and incubating (>1 h) in serum-free medium+10 mM HEPES immediately before use. Stack B is made by placing a dry hydrophobic spacer (18×18 mm; ‘Rite in the Rain’ paper) with a central square hole (6×6 mm) in the middle of the wetted octagon. Although medium slowly wicks upwards into this spacer (the spacer adheres firmly to the underlying octagon as it does so), flow through the spacer will be low (as a rate of only ∼4.3 µl/h is obtained when a square of ‘Rite in the Rain’ paper without a hole replaces the agarose-containing regulator in stack A in an experiment like that illustrated in Fig. 6A). Three squares containing cells are stacked centrally (with creped ridges co-aligned) over the ‘Rite in the Rain’ spacer followed by a separating cell-free filter paper 2 (Whatman number 1, 10×10 mm) wetted with medium, and the stack capped with a stainless-steel washer (O.D. 8 mm, I.D. 4.3 mm, height 5 mm). Paper 2 is added to provide a flat interface between the capping washer and the top-most crepe paper containing cells; however, when omitted on other occasions this had no discernible effect on experimental outcomes. Washers capping all five stacks are wet by splitting medium (100 µl) between them, and sterile FC40 (pre-saturated with water) added half-way up the washer on top of stack B. Such FC40 is prepared by autoclaving a glass bottle containing a stainless-steel cloning ring (OD 8 mm, ID 6 mm, height 10 mm) stuffed with filter paper, pipetting 200 µl PBS on to the paper, adding 20 ml sterile FC40, and incubation (>18 h; room temperature). Cells are fed twice daily (using medium stored at 37°C from day 1, so control and stacked cells receive medium exposed to the same thermal conditions) for up to 10 days by pipetting 50 µl/input stack and removing 200 µl from stack B. [As inputs of 4×50 µl are pipetted into stacks A1 to A4 over ∼1 min, and as caps on input washers disappear in minutes, some medium probably flows wastefully around modules as well as through them (confirmed using dyes in Fig. S7A); therefore, further research is needed to determine how much waste occurs, and how it might be mitigated (e.g. by developing better regulators, as in Fig. 7B).] If excess medium buds off from stack B to float to the surface, it is removed by pipetting. Finally, papers are stained and imaged as on day 1.

For Fig. S4, a filter paper (Whatman number 50, 30×10 mm, ∼100 µm thick, particle retention 2.7 µm; this paper was chosen because it retains smaller particles than Whatman number 1 paper, marginally slows flow through it, and acts as a pre-filter to remove large serum aggregates) is placed in a 60 mm dish. Next, input stack A is built by adding successively a Millipore MF 0.1 µm pore VCWP filter disc (diameter 13 mm), a pre-filter (11×11 mm square of Whatman number 50 paper with corners removed so it is ∼1 mm wider than 10 mm in all dimensions), and a washer (OD 10 mm, ID 5 mm, thickness 1 mm). Output stack B is built by adding one tall washer (OD 8 mm, ID 4.3 mm, height 5 mm). Medium+10 mM HEPES (150 µl) is now pipetted into stack B; it wicks through the paper to wet the filter and pre-filter in stack A. After overlaying FC40 (18 ml), medium is added to stack B until a spherical cap just becomes visible. Over the next 24 h, 175 µl medium+blue dye (0.5 mg/ml resazurin) is pipetted manually in 25 or 50 µl aliquots into stack A; it flows to stack B, and is then removed by pipetting. Images are collected from the side at intervals. FC40 is removed, and stack A dismantled and rebuilt by replacing the filter with 0.1 µm pores with another containing 0.2 µm pores (Millipore Isopore PC membrane, 13 mm diameter; pre-wetted with medium without serum or dye). Next, a fresh pre-filter like the one used before (also pre-wetted with medium without serum or dye) is added, followed by the same washer used previously. Then, FC40 (18 ml) is overlaid, medium minus dye added to stack B until a cap becomes just visible, aliquots of medium plus blue dye pipetted into stack A, images collected, and flow rates calculated (averages of 11 successive pairs of images for 0.1 µm pores, and four pairs of images for 0.2 µm pores). Flow remains essentially constant during the 54 and 4 min of these parts of the experiment. However, flows slow over hours, presumably as components in serum clog pores. As the ID of the washer in stack B is smaller than that in stack A, differences in Laplace pressure in the two spherical caps tend to limit flow driven mainly by hydrostatic pressure.

For Fig. S5A, a circuit with two inputs is made by laying a filter-paper rectangle (40×10 mm; Whatman number 1) on a 60 mm dish, building stacks with one or three stainless-steel washers (OD 10 mm, ID 5 mm; thickness 1 mm; hole volume ∼20 µl), wetting the paper with 100 µl PBS, overlaying 15 ml FC40, and pipetting 100 µl through stack B on to the paper to give a cap. Images are then collected before (0 min) and after pipetting 25 µl aliquots of PBS plus blue or red dye (200 µg/ml resazurin, or 400 µg/ml Allura Red) through stacks A and C respectively on to the underlying paper (two aliquots added in the first minute, and one every minute thereafter).

The circuit in Fig. S5B with multiple but connected outputs is built and operated as follows. The challenge here is to ensure that stacks B–D all have the same pressure difference from top to bottom; this is achieved by interconnecting outputs through a bridge. The circuit is built in a large non-tissue-culture-treated dish (Sarsted; 92×16 mm; ThermoFisher Scientific) containing a filter-paper rectangle (80×10 mm, Whatman number 1) wetted with 100 µl PBS. Two agarose blocks cast in PBS (each 10×10 mm, ∼2.5 mm thick) and a stack of 12 filter-paper squares (10×10 mm, prewetted with PBS) are then placed on the filter paper (with centers positioned 20 mm apart along the paper). Next, two hydrophobic spacers (PTFE washers, OD 13 mm, ID 6 mm, thickness 0.55 mm) plus a hydrophilic washer (stainless-steel, OD 10 mm, ID 5 mm, thickness 1 mm) are added coaxially to stacks B–D, and annuli filled with a small ball of cotton wool (pre-wetted with PBS) that will provide vertical aqueous continuity in the stack. FC40 (5 ml) is added to prevent PBS spreading across the dish. After adding 100 µl PBS to each cotton-wool ball, a filter-paper rectangle (50×10 mm, Whatman number 1) pre-wetted with 100 µl PBS is laid over the three growing stacks, then a PTFE spacer (OD 13 mm, ID 6 mm, thickness 0.55 mm), and finally an M6 nut (width across flats 10 mm; thickness 5.2 mm) as a weight. Next, FC40 is added up to the level of the topmost PTFE washer in stack D (which happens to be slightly higher than its counterparts in stacks B and C). Outputs from stacks B–D share a common high point in stack D, as they inter-connect through the upper filter paper (shown horizontal in the cartoon in Fig. S5Ai); therefore, stacks B–D have roughly the same pressure difference from top to bottom. A dispensing needle (inserted in a holder and connected to a syringe pump) is lowered on the bottom filter paper 10 mm from the left-hand end. Images are then collected as blue dye (50 µl resazurin in PBS) is deposited (at 2 µl/min) on to the paper. After 6 h, flow is increased to 4 µl/min.

For Fig. S6A (where HEKs are grown in a silk fibroin mesh), mesh squares (10×10 mm, thickness 211 µm) cut from the roll provided by the supplier proved to be curved, rigid, difficult to stack, and wetted poorly with Geltrex – so individual chambers are difficult to fill with liquid hydrogel. Therefore, an attempt was made to increase hydrophilicity by oxidation by the Fenton reaction using H_2_O_2_ and Fe^2+^. In the first experiment, the reaction destroyed many chambers, but the control without Fe^2+^ yielded flexible and stackable meshes with slightly increased hydrophilicity; therefore, meshes are treated as follows. Two meshes (each 150×87.5 mm) are incubated (60°C, overnight) in a 50 ml centrifuge tube filled with equal volumes of PBS and H_2_O_2_ (supplied as a 30% solution; a needle hole in the lid allows gas escape), meshes rinsed thoroughly in distilled water, air dried at room temperature, and cut into 10×10 mm squares. Meshes are filled with Geltrex as follows. The manufacturer recommends setting liquid ice-cold hydrogels by incubation at 37°C for 30 min; however, here we set gels by incubation in FC40 at 37°C, but pilot experiments showed a 30 min incubation reduced cloning efficiency (presumably because too much evaporation occurs from the nanoliter volume in each mesh chamber). Therefore, gelling time is reduced to 10 s, and FC40 is pre-saturated with water. Thus, 1 volume HEKs in medium (10^6^/ml)+10 mM HEPES is mixed with nine volumes of ice-cold Geltrex+10 mM HEPES in a cryogenic vial (1.8 ml, Star Lab Ltd, Milton Keynes, UK) – chosen only because it is a small tube with a 10 mm diameter and so the width of the mesh. In contrast to the lower Geltrex concentration used in Fig. 7, this one is sufficient to form a free-standing gel. Thus, each mesh square is picked up with tweezers, dipped into Geltrex+cells (in a dish on ice) for ∼10 s to load mesh chambers with liquid gel, then into water-saturated FC40 (37°C, 15 s) to set the gel, and dropped into a Petri dish containing medium at 37°C. Saturated FC40 is prepared in this case by adding three squares (10×10 mm) of Whatman number 113 filter paper to a cryovial followed by a washer, squashing the paper with the washer, pipetting 200 µl PBS on to the paper, filling the vial with FC40, sterilizing by autoclaving, and incubating at 37°C for >3 h. Meshes submerged in medium are then incubated for times indicated.

For Fig. S6B (where HEKs are grown in both unstacked and stacked nylon meshes), a sheet of monofilament nylon 6.6 mesh (thickness 340 µm, mesh opening 400 µm, 43% open area; Cadisch Precision Meshes, Hertfordshire, UK) is cut into 10×10 mm squares, each square picked up with tweezers, dipped successively in ice-cold Geltrex+cells (in a dish on ice) for ∼10 s (to load mesh chambers with liquid gel) and then water-saturated FC40 at 37°C for 10–15 s (to set the gel), immediately dropped into medium+10 mM HEPES at 37°C, and unstacked meshes incubated overnight (all as for Fig. S6A). Next day, the stack is constructed (from bottom up) on a filter paper (10×12 mm; Whatman number 1) with a central peephole (5 mm) and pre-wetted with medium, 5×nylon meshes (stacked centrally on the filter-paper base), a spacer (12×12 mm; ‘Rite in the Rain’ paper pre-wetted with FC40) with a central peephole (5 mm), and a stainless-steel tube as weight (O.D. 8 mm, I.D. 4.3 mm, height 5 mm). FC40 is added up to the level of the spacer, medium added to fill the tube acting as a weight, and more FC40 added to cover the top of the tube. Stacks are fed by pipetting medium on to the filter-paper base and removing an equal volume from the cap on top of the stack. All medium used to feed both unstacked and stacked meshes is stored at 37°C from the beginning, so control and stacked meshes are fed with medium exposed to the same thermal conditions. In principle, cloning efficiencies and clonal volumes can be calculated in much the same way as one does using a hemocytometer, but various factors make determining both challenging. These include: (1) inevitable complications due to 3D imaging (e.g. some cells/colonies are out-of-focus), (2) local variations in gel thickness (gels appear detectably thinner in the microscope in the middle of ∼20% mesh chambers, with another ∼5% having obvious central holes in the gel), and (3) colony shape (internal colonies are usually spherical, but cells grow as extensive monolayers on surfaces, and colonies growing between meshes may be ripped apart or lost during unstacking). Nevertheless, we estimate a rough cloning efficiency as follows. Consider the volume under an area of 400×400 µm in the image in Fig. S6B after 14 d. Assuming the gel is 340 µm thick, ∼5 single cells are initially seeded in this volume, and ∼2 colonies are detected. Then, the cloning efficiency is close to the ∼50% obtained conventionally (Soitu et al., 2020a,b). In practice, we think counting colonies by eye as one focuses up and down through the volume of Geltrex within one chamber (i.e. much as one does when using a hemocytometer) is more accurate than counting them in images.

For Fig. S7A, the circuit is built like the one in Fig. 7 with the following exceptions. First, 3 Whatman number 113 filter papers in central stack B are filled with cell-free Geltrex – as opposed to cell-containing Geltrex. Second, 20 ml FC40 is overlaid (in Fig. 7, slightly less FC40 is added). Third, the experiment is conducted at room temperature in a lab (not a CO_2_ incubator). Fourth, blue dye (25 µl; 0.5 mg/ml resazurin DMEM+10 mM HEPES) is pipetted into each of A1–A4 at *t*=0, 15, 30, and 45 min (not twice daily); at *t*=60, the output reservoir is emptied, all FC40 removed, and the stack dismantled.

For Fig. S7B, the central stack B above the flow regulator is as in Fig. S7A; however, dish type (here treated for tissue culture, and not untreated), paper 1, the sole flow regulator, and input reservoirs all differ. Paper 1 is a circular disc of Whatman number 50 paper (diameter ∼45 mm, thickness ∼100 µm, particle retention 2.7 µm); this is smoother, thinner, and has smaller pores than Whatman number 1 (with thickness ∼180 µm, particle retention 11 µm). The flow regulator is a pre-wetted disc of hydrophobic ‘Rite in the Rain’ paper (diameter ∼47 mm) with a central hole made with a hole punch (diameter 4 mm). The four input reservoirs (A1–A4) are 3D printed (as one connected structure) using a FlashForge Finder (Flashforge 3D Technology, Zhejiang, China; 0.4 mm nozzle) and white poly-lactic acid (PLA; filament diameter 1.75 mm). The structure consists of two concentric circular rings (each 1.2 mm wide, 2 mm high) separated by a 1.5 mm gap; rings are held in position by four radial struts that divide the gap into four equally sized quadrants (input reservoirs A1−A4). Radial distances from ring center to inner and outer walls of the inner ring, are 17.5 and 18.7 mm, respectively; corresponding distances to inner and outer walls of the outer ring are 20.2 and 21.4 mm, respectively. Axial struts run from the inner wall of the inner ring to beyond the outer wall of the outer ring (total strut length 8 mm); each is 3 mm wide, and 1 or 2 mm high (between outer and inner rings, or projecting beyond the outer ring, respectively). The structure fits loosely into a 60 mm dish (inner diameter at the bottom is 51.8–51.9 mm). PLA is hydrophilic, and the printed structure is inverted for use so the bottoms of axial projections lie 1 mm above the hydrophobic regulator (to limit medium wicking from reservoirs to the edge of the dish). The circuit is made by placing 400 µl medium+10 mM HEPES in the middle of a dish, overlaying paper 1 followed by the regulator. Once medium has wicked throughout the hydrophobic regulator, medium (100 µl) is pipetted into the hole in the regulator, and the stack built by placing three filter-paper squares (Whatman number 113; 10×10 mm) pre-wetted with medium over the hole, followed by one pre-wetted paper 2 (Whatman number 1; 10×10 mm), and the stack capped with a stainless-steel washer (O.D. 8 mm, I.D. 4.3 mm, height 5 mm). Next, reservoirs A1-A4 are each filled with 100 µl medium+10 mM HEPES (here, we fill only up to the level of the reservoir wall to limit medium loss by floatation above the FC40). Now, 1 ml FC40 is pipetted onto the bottom of the dish between the dish wall and the paper circles to prevent outward wicking of medium, the output reservoir on stack B filled with medium+10 mM HEPES (100 µl), and the dish filled with 19 ml FC40. After equilibration (15 min), the volume in the reservoir on stack B is adjusted so medium just fills the washer to the top when viewed from the side. At *t*=0, 25 µl blue dye (0.5 mg/ml resazurin in DMEM+10 mM HEPES) is pipetted into each input quadrant. At *t*=5 h, 140 µl is removed from the cap on stack B to restore the medium level so it again just fills the washer (indicating a flow rate of 140 µl/5 h); additionally, a total of 350 µl is added to the four quadrants. At *t*=20 h, 400 µl is removed from the cap to restore the medium level so it again just fills the washer (indicating a flow rate of 400 µl/15 h). At *t*=20 h, the output reservoir is emptied, all FC40 removed, and the stack dismantled. While we still seek better regulators and base papers, the ones used here are currently those of choice. Comparison of flows through an identical circuit with wider input quadrants (i.e. 2 mm) increases reservoir capacity and yields a higher flow rate (i.e. ∼235 µl/5 h). As PLA has a density of 1.24 g/cc, increasing reservoir width beyond 2 mm requires use of weights to prevent them from floating on FC40. Note also that PLA softens at ∼50°C, and so is sterilized using 70% ethanol (not by autoclaving).

For Fig. S8, conditions are as described for Fig. 7 with the following exceptions. (1) Stacks are prepared on dishes treated for tissue culture (Corning number 430196, 60 mm×15 mm style, tissue culture treated) as suspension dishes became unavailable during that stage in the COVID-19 pandemic (this difference had no observed consequences during operation or on outcome), the central stack B contains five papers instead of three, and this stack is fed three times daily from days 6–9 (using medium from a tube stored in the incubator for the first addition, and by recycling medium from the cap on stack B into input stacks A1–4 for the second and third). (2) For the stack of three papers, no medium was added on days 2–9 after filling the capping weights in stacks A1–A4 and on stack B to check aqueous continuity in the circuit on day 1 (Fig. S8v).

### Challenges associated with determining cloning efficiencies, growth rates, and degree of necrosis

In principle, cloning efficiencies and growth rates can be calculated using images collected in the experiments illustrated in Fig. 7 and Fig. S8; in practice, various factors make determining doing so challenging. These include: (1) inevitable complications due to 3D imaging (e.g. poor depth of focus of ∼10 µm for a 10× objective with numerical aperture of 0.25, and out-of-focus flare), (2) almost two-fold local variations in thickness of the filter paper that is creped (thickness varies between ∼240−440 µm), coupled to additional local variations in paper density (Fig. 7i, phase-contrast image), (3) penetrance rate of calcein AM through paper, Geltrex, and peripheral cells in colonies, (4) colony shape (colonies often fuse and/or adopt variable shapes defined by paper pores/surfaces, and some growing between papers are ripped apart or lost during unstacking), and (5) light scatter (note significant fluorescent halos around all fluorescent colonies in images). Against this background, we estimate a rough cloning efficiency as follows. Consider the volume under an area of 400×400 µm in the image in Fig. 7 of stack 2 after 10 days. Assuming the paper is 340 µm thick, the seeding solution contains ∼5 single cells in a volume of 400×400×340 µm, and ∼1.5 colonies are detected. As paper occupies some volume, the cloning efficiency is then close to the ∼50% obtained conventionally (Soitu et al., 2020a,b). In practice, we think counting deposited cells or colonies by eye as one focuses up and down through the volume of paper in various microscopic fields of view is more accurate than counting them in images. For example, our 10x objective has a circular field of view (diameter 2.1 mm), and we can focus up and down through a volume of ∼1.1 µl in one paper to count fluorescent spots or halos. In Fig. 7, an unstacked control on day 1 contained an average of 49 small fluorescent spots/halos in the central 10 fields (range 39–58). [In similar experiments, averages are 51 (range 41–57), 33 (range 28–45), 52 (range 47–61), and 42 (range 37–47).] As 110 cells are present per 1.1 µl in the Geltrex-containing seeding solution in this experiment, and if all spots/halos are detected and each one represents a single cell, we detect 49 spots/cells per field of view (presumably some cells are missed, and/or paper occupies some volume). Counting colony numbers on day 10 is even more challenging as the scatter of light emitted by fluorescent colonies is so significant, as colony shape is so variable, and as many colonies merge. Nevertheless, the density of the now-larger spots/halos appears much the same as on day 1. Therefore, we conclude the cloning efficiency is within the range obtained conventionally. Volume of clones are also (roughly) estimated as follows (Fig. 8A). Using fluorescence images of the 10 different fields in the middle of papers on day 1 and day 10, the most in-focus cells/colonies are selected (usually just ∼2/field), and fluorescence areas determined using ImageJ. We assume a fluorescent focus seen on day 1 contains one cell (but some cells might have divided since seeding the previous day), and that colonies on day 10 completely fill a spherical colony (but Fig. 7 and Fig. S8 shows colonies are often non-spherical, and some paper fills the volume). Then, cell and clone volumes are calculated assuming both are paper-free spheres. Additionally, we test whether clones in stacks contain necrotic cores (Fig. 8B) as follows. As stated previously, the red-fluorescent ethidium homodimer-1 (provided in the LIVE/DEAD Viability/Cytotoxicity kit to detect dead cells) binds to paper to give unusably high backgrounds, so we could not use it here. Using the same images of in-focus cells/colonies described above, we collect a line scan (1 pixel wide) across the longest axis of each colony, and across the orthogonal axis (using ImageJ). We also assume colonies with a central necrotic core should have central valleys in both profiles (Fig. 8Bi), and score a colony as ‘possibly necrotic’ if the intensity anywhere at the middle two-thirds of both scans is <37% peak value (this is the expected value if 25% volume at center of a sphere is non-fluorescent due to necrosis).

## Supplementary Material

10.1242/biolopen.059825_sup1 Supplementary information
