# Supplementary material for: A platform for modular assembly and feeding of micro-organoids on standard Petri dishes
Source: Biol Open. 2023 May 19;12(5):bio059825. doi: 10.1242/bio.059825 (PMC10214846; doi:10.1242/bio.059825)
Supplement: Supplementary information [file biolopen-12-059825-s1.pdf]

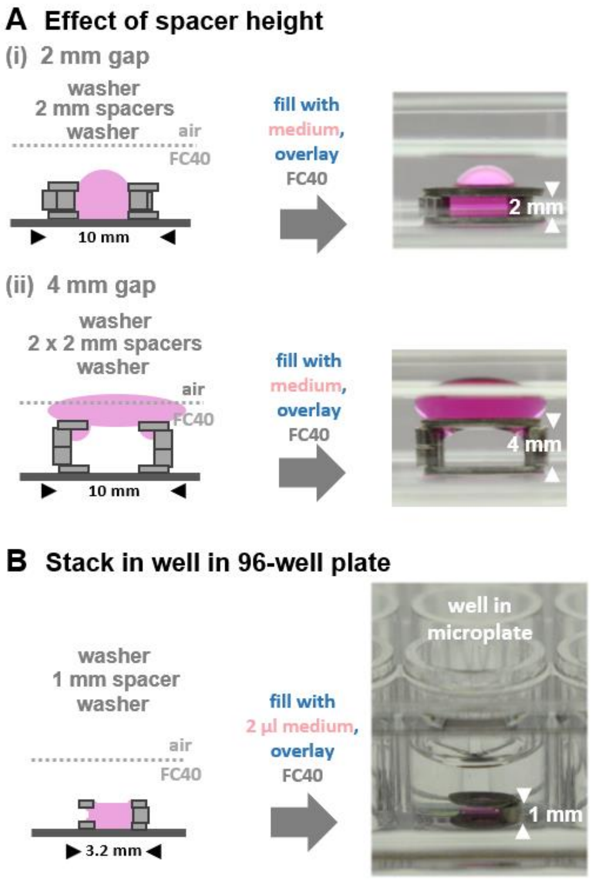

Fig. S1. The stability of liquid columns in stacks of stainless-steel washers.

A. Introducing tall spacers into stacks destabilizes fluidic towers. Stacks are assembled using two washers (OD 10 mm, ID 5 mm, thickness 1 mm, washer hole ~20 µl) separated by tube magnets/spacers (width 1 mm, height 2 mm) to create gaps of 2 or 4 mm high, stacks filled with medium, FC40 overlaid, and images collected.

(**i**) A stack with a 2 mm gap is stable if the dish is carried carefully, as interfacial forces pinning medium to the dish are stronger than buoyancy forces driving medium above denser FC40.

(**ii**) A stack with a 4 mm spacer is unstable, and medium spontaneously detaches from the dish but remains attached to the topmost washer. If the dish is shaken, the cap buds off to float on the FC40.

B. Miniature stack in a well in a 96-well plate. The stack contains two M1 washers (OD 3.2 mm, ID 1.1 mm, thickness 0.3 mm, hole volume ~0.3 µl) separated by a tube magnet/spacer that is 1 mm high and 1 mm thick. After placing the stack in a well in a 96-well plate, 2 µl medium is pipetted through holes in the two washers on to the bottom of the well, FC40 overlaid, and the well imaged. Medium remains pinned to the bottom and retained in the stack.

Biology Open • Supplementary information

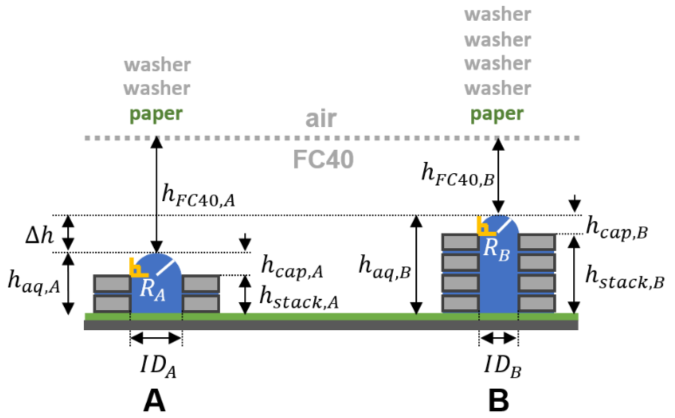

Fig. S2. Defining flow direction in a simple circuit (stack A has two washers, stack B has 4 washers, and both sit on a pre-wetted filter paper. Flow between stacks is driven by pressure differences generated by hydrostatic heads of aqueous and fluorocarbon phases, plus Laplace pressure. Pressures at the base of the aqueous towers in stacks A and B (P_A and P_B) are:

$$P_A = \rho_{FC40} g h_{FC40,A} + \rho_{aq} g h_{aq,A} + \frac{2\gamma}{R_A} \quad \text{S1}$$

$$P_B = \rho_{FC40} g h_{FC40,B} + \rho_{aq} g h_{aq,B} + \frac{2\gamma}{R_B} \quad \text{S2}$$

where ρ_{FC40} and ρ_{aq} are densities of the two phases, g represents gravitational acceleration (9.81 m^2/s), γ is the interfacial tension (23 mN/m; Deroy et al., 2021), and all heights and radii are defined in the Figure. If $P_A > P_B$, flow is to the right; if $P_A < P_B$, it is to the left. Given Eq.S1 and Eq.S2, one gets:

$$P_B - P_A = \rho_{FC40} g h_{FC40,B} + \rho_{aq} g h_{aq,B} + \frac{2\gamma}{R_B} - \left(\rho_{FC40} g h_{FC40,A} + \rho_{aq} g h_{aq,A} + \frac{2\gamma}{R_A}\right)$$
$$\therefore P_B - P_A = \rho_{FC40} g \left(h_{FC40,B} - h_{FC40,A}\right) + \rho_{aq} g \left(h_{aq,B} - h_{aq,A}\right) + 2\gamma\left(\frac{1}{R_B} - \frac{1}{R_A}\right) \quad \text{S3}$$

From the Figure one gets:

$$\Delta h = h_{FC40,A} - h_{FC40,B} = h_{aq,B} - h_{aq,A}$$
$$\therefore h_{FC40,B} - h_{FC40,A} = -\left(h_{aq,B} - h_{aq,A}\right) \quad \text{S4}$$

Substituting in Eq. S3 gives:

$$P_B - P_A = -\rho_{FC40} g \left(h_{aq,B} - h_{aq,A}\right) + \rho_w g \left(h_{aq,B} - h_{aq,A}\right) + 2\gamma\left(\frac{1}{R_B} - \frac{1}{R_A}\right)$$
$$\therefore P_B - P_A = -\left(\rho_{FC40} - \rho_{aq}\right) g \left(h_{aq,B} - h_{aq,A}\right) + 2\gamma\left(\frac{1}{R_B} - \frac{1}{R_A}\right) \quad \text{S5}$$

If $\Delta P = P_B - P_A = 0$, the system is in equilibrium, and no flow occurs. Therefore

$$-\left(\rho_{FC40} - \rho_{aq}\right) g \left(h_{aq,B} - h_{aq,A}\right) + 2\gamma\left(\frac{1}{R_B} - \frac{1}{R_A}\right) = 0 \quad \text{S6}$$

and

$$\left(h_{aq,B} - h_{aq,A}\right) = \frac{2\gamma\left(\frac{1}{R_B} - \frac{1}{R_A}\right)}{\left(\rho_{FC40} - \rho_{aq}\right) g} \quad \text{S7}$$

As

$$h_{aq,A} = h_{stack,A} + h_{cap,A} \qquad \text{S8}$$
$$h_{aq,B} = h_{stack,B} + h_{cap,B} \qquad \text{S9}$$

Eq. S7 becomes:

$$\left(h_{stack,B} - h_{stack,A}\right) = \frac{2\gamma\left(\frac{1}{R_B} - \frac{1}{R_A}\right)}{(\rho_{FC40} - \rho_{aq})g} + h_{cap,A} - h_{cap,B} \qquad \text{S10}$$

Finally, for the sake of simplicity, we assume both spherical caps always have contact angles of 90° (which gives the maximum Laplace pressure for a particular internal diameter), so that both cap heights and radii of curvature equal inner radii (half of inner diameters ID_A and ID_B) of the washers. Then:

$$h_{cap,A} = R_A = \frac{ID_A}{2} \qquad \text{S11}$$
$$h_{cap,B} = R_B = \frac{ID_B}{2} \qquad \text{S12}$$

This leads to the equilibrium equation that depends solely on user-defined variables (stack heights and internal diameters), plus known constants (γ, ρ_{FC40}, ρ_{aq}, g):

$$\left(h_{stack,B} - h_{stack,A}\right) = \frac{4\gamma\left(\frac{1}{ID_B} - \frac{1}{ID_A}\right)}{(\rho_{FC40} - \rho_{aq})g} + \frac{ID_A - ID_B}{2} \qquad \text{S13}$$

Eq. S13 represents a useful tool when designing circuits. It relates pressures in two fluidly-connected stacks while neglecting the path between them, and without quantifying flow rate. It enables biologists to answer the practical question: what are the differences in stack height and internal diameters of input and output stacks (two variables under the user's control) that enables one to be sure that flow will be in the desired direction (see **Figure 3**).

Biology Open • Supplementary information

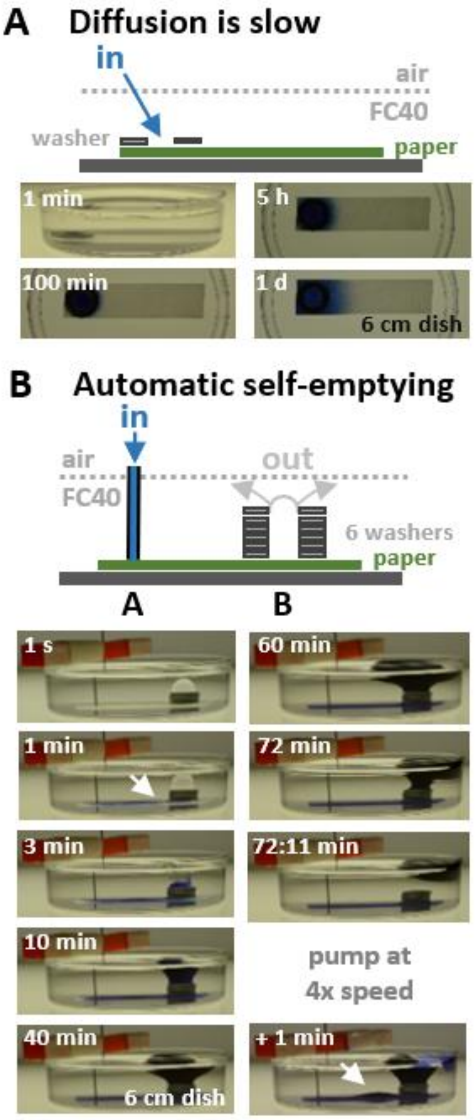

Fig. S3. While diffusion through filter-paper (Whatman No 1) is slow, flows can be driven rapidly (by an external pump) through paper, and up a stack to an output reservoir that can spontaneously self-empty (60 mm dishes).

A. Diffusion is slow. The cartoon illustrates the structure built by placing a paper rectangle (40 x 10 mm) in a dish followed by a steel washer (OD 10 mm, ID 5 mm, thickness 1 mm) at the left-hand end (which acts as a weight), wetting the paper with 75 µl PBS, and overlaying 20 ml FC40. Next, 5 µl blue resazurin dye (4 mg/ml) is pipetted manually through the hole in the washer onto the paper. Images show side and top views. After 1 d, dye has yet to reach the middle of the strip (which is as expected, as the diffusion coefficient of the dye is 4.8×10^{-6} cm^2/s; Fan et al., 2018).

B. High flows and automatic self-emptying. The cartoon illustrates the structure, which is built by placing a paper rectangle (30 x 10 mm) in a dish, stacking 6 M3 washers (OD 7 mm, ID 3.1 mm, thickness 0.5 mm) at the right-hand end, wetting the paper with 50 µl PBS, pipetting 100 µl PBS into the stack to wet washers, and overlaying 20 ml FC40. PBS initially spills out of the stack to puddle on the surface of the filter paper, but the system rapidly equilibrates once FC40 is added and puddle contents are sucked into the cap on

Biology Open • Supplementary information

stack B. Next, a stainless-steel needle – filled with PBS + blue dye (50 µg/ml) and connected to a syringe pump – is lowered on to the filter paper. Starting the pump delivers dye (25 µl/min) onto the paper (this flow rate is chosen as it is just below the maximum the system can sustain without forming puddles on the surface of the paper), and then flow is to the point of lowest pressure (the cap on stack D). Stills taken from a movie illustrate resulting changes in fluid structure. The dye front almost reaches stack B after 1 min (arrow), and by 3 min it enters the cap. After 10 min the cap has filled with dye and expanded sufficiently to reach above the FC40 surface. Over the next hour, the cap continues expanding and eventually touches the side of the dish (at 72 min); this destabilizes the structure, and the cap buds off from stack (at 72:11 min) to float on the surface. The pump is stopped, and most of the now-floating cap removed by pipet (some remains at the side). The pump is now restarted at 100 µl/min, to illustrate what happens when input volume exceeds the capacity of the system to suck fluid into the cap. Although the cap continues to expand, some dye puddles on the surface of the paper (+1 min, arrow). Subsequently, this puddle expands and eventually rises up the outer edge of the stack to float on the FC40 (not shown).

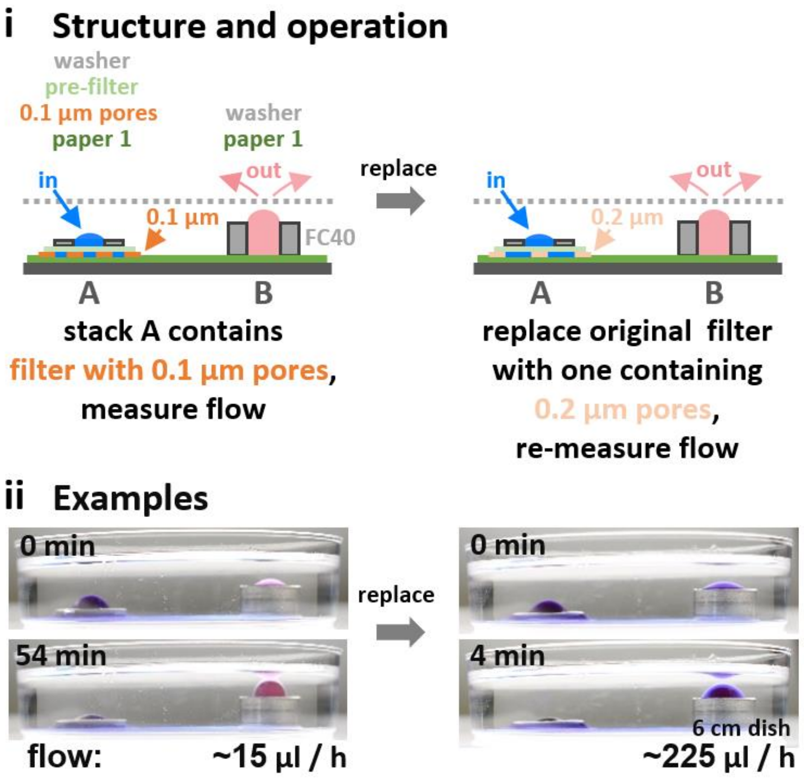

Fig. S4. Using Millipore^{TM} filters as flow regulators (60 mm dish). Flow rates are calculated as in Figure 6.

(**i**). Structure and operation. Initially, stack A contains a Millipore filter with 0.1 µm pores that limits flow (plus a paper pre-filter to remove any aggregates from serum that might block pores in the Millipore filter), stack B consists of one tall washer, and the circuit is filled with medium (pink). Medium + blue dye are manually pipetted into stack A so the front of blue dye first reaches the cap on stack B; then, more dye is pipetted into stack A to create a spherical cap, images are collected as the cap shrinks, and the flow rate is calculated. FC40 is now removed, and stack A dismantled and rebuilt except the original filter is replaced with one containing 0.2 µm pores. After pipetting more dye into stack A to create a cap, the new flow rate is determined.

(**ii**). Example images and flow rates before (left) and after replacing one Millipore filter with another (right). As expected, flow through the filter with large pores is faster.

Biology Open • Supplementary information

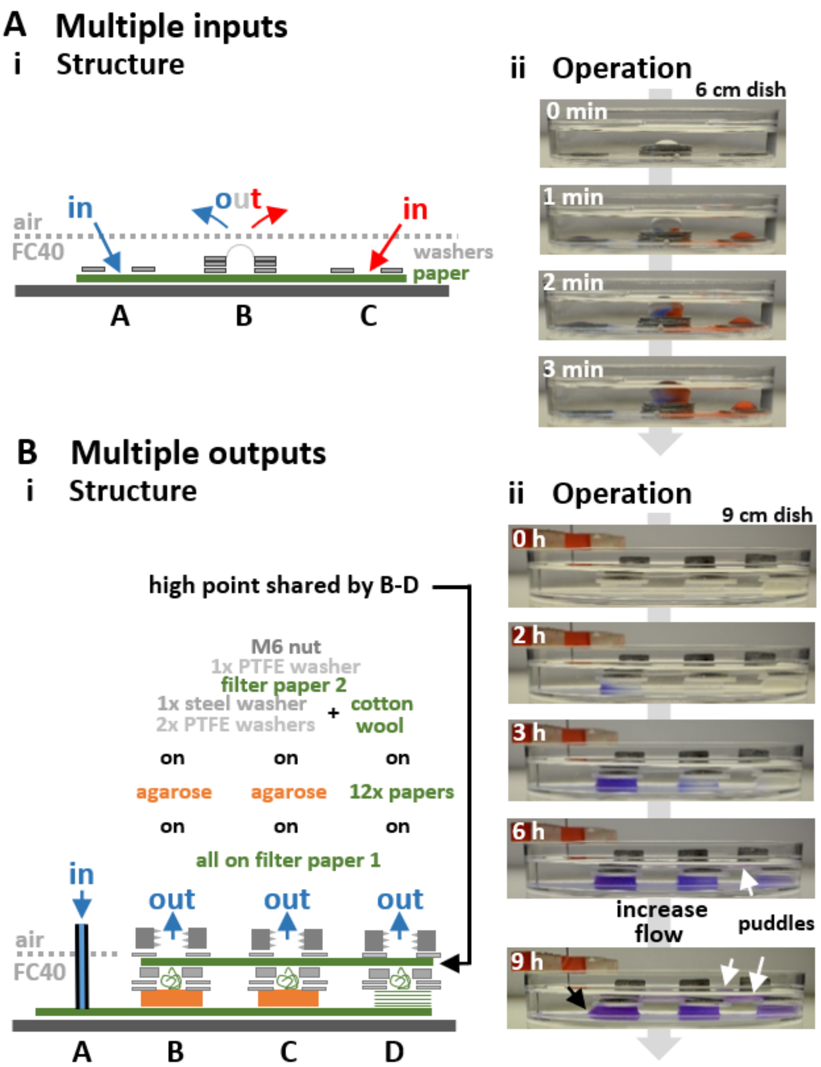

Fig. S5. Circuits with multiple inputs and outputs.

A. Multiple inputs. (**i**) Structure (60 mm dish). Stacks A and C are inputs and B is the output. (**ii**) Images collected before (0 min) and after pipetting 25 µl aliquots of blue and then red dye (resazurin or Allura Red) into stacks A and C on to the underlying filter paper (two aliquots added in the first minute, and one thereafter ever minute). Differences in hydrostatic pressure are the main driver of flows into the cap on stack B where the two dyes mix.

B. Multiple outputs. The highest point in an aqueous circuit has the smallest hydrostatic head of FC40 above it, and so the lowest pressure (neglecting effects of Laplace pressure). However, it is difficult to build multiple high points at exactly the same level, and then the highest dominates to suck fluid from the others. Here, outputs from stacks B-D are connected so they share a common high point (the upper filter paper). (**i**) Structure (9 cm dish). Stacks B and C each contain an agarose block, and D a stack of filter-paper squares. Each module sits on filter-paper 1; blue dye is delivered on to this at A. Each module is overlaid with identical stacks of washers (some filled with cotton wool) that enable aqueous connection with an upper filter paper 2. Outputs from stacks B-D share a common high point, as they are inter-connected through filter paper 2; consequently,

Biology Open • Supplementary information

stacks B-D have the same pressure difference from top to bottom as their highest points are inter-connected. (**ii**) Operation. Over time, images show dye flowing laterally and vertically through the system to accumulate in puddles around the upper filter paper (white arrows). The flow rate is doubled after 6 h; as this slightly exceeds the capacity of the system, some dye puddles around the bottom of the agarose block in stack B (black arrow).

Biology Open • Supplementary information

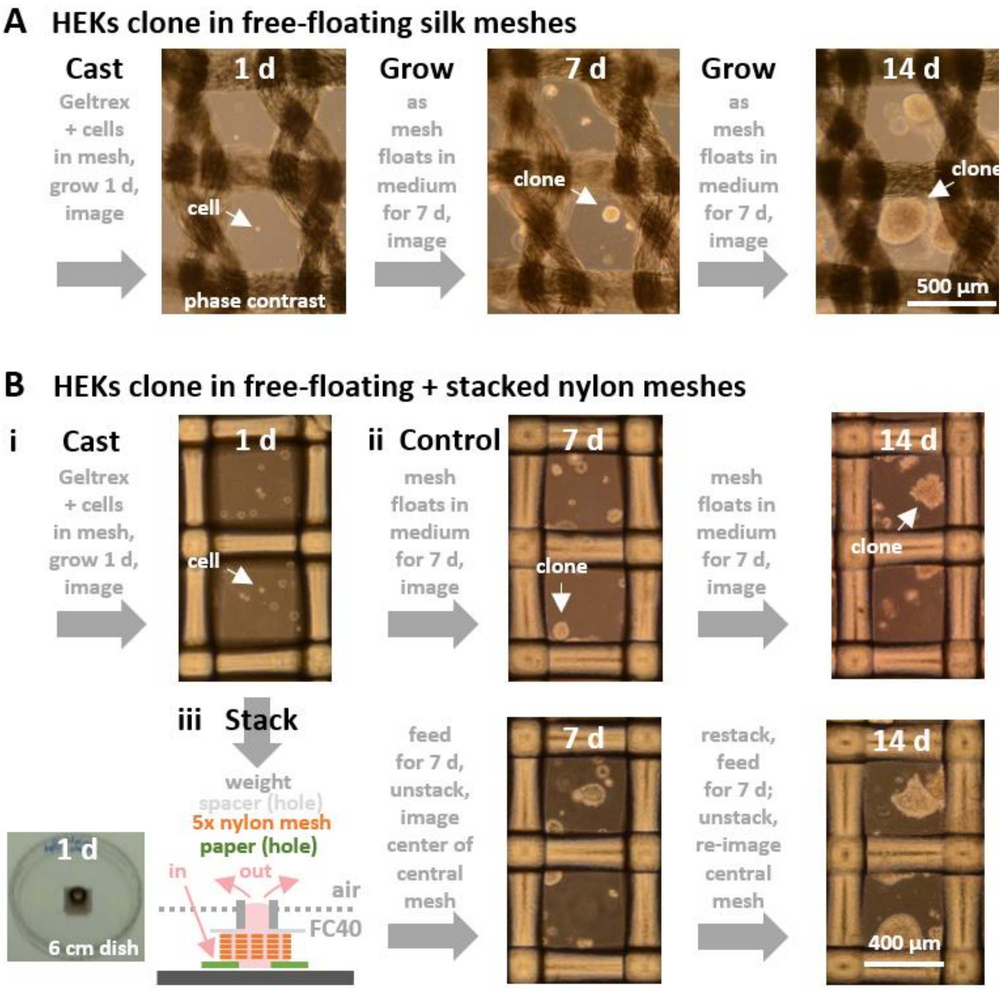

Fig. S6. HEK clones grow as expected in Geltrex in silk and nylon meshes. Square meshes (10 x 10 mm) are dipped successively in ice-cold and liquid Geltrex containing HEKs so each mesh chamber fills with liquid and a few cells, and gels are set by incubation at 37°C (for 15 s in FC40). Then, meshes are transferred to medium or incorporated into stacks, and phase-contrast images of typical central regions of squares collected at the times indicated (no attempt is made to image the same openings).

A. Unstacked silk meshes (~457 µm openings, thickness ~211 µm) immersed in medium. Cells are fed by diffusion.

B. Unstacked and stacked nylon meshes (openings 400 x 400 µm; thickness ~340 µm).

(**i**) Cells are cast in Geltrex, and grown as unstacked squares immersed in medium for 1 d.

(**ii**) As controls, cells in unstacked squares are grown for 7 and 14 d as colonies develop (fed by diffusion).

(**iii**) A stack of 5 squares is prepared on day 1 (cartoon shows structure, with image of the completed stack on the left). Subsequently, cells in the stack are fed by pipetting medium on to the filter-paper base and removing an equal volume from the cap on the stack; transfer is driven by a difference in hydrostatic pressure between the top and bottom of the stack. Colonies develop with cloning efficiencies much like those in the control (see Materials and Methods).

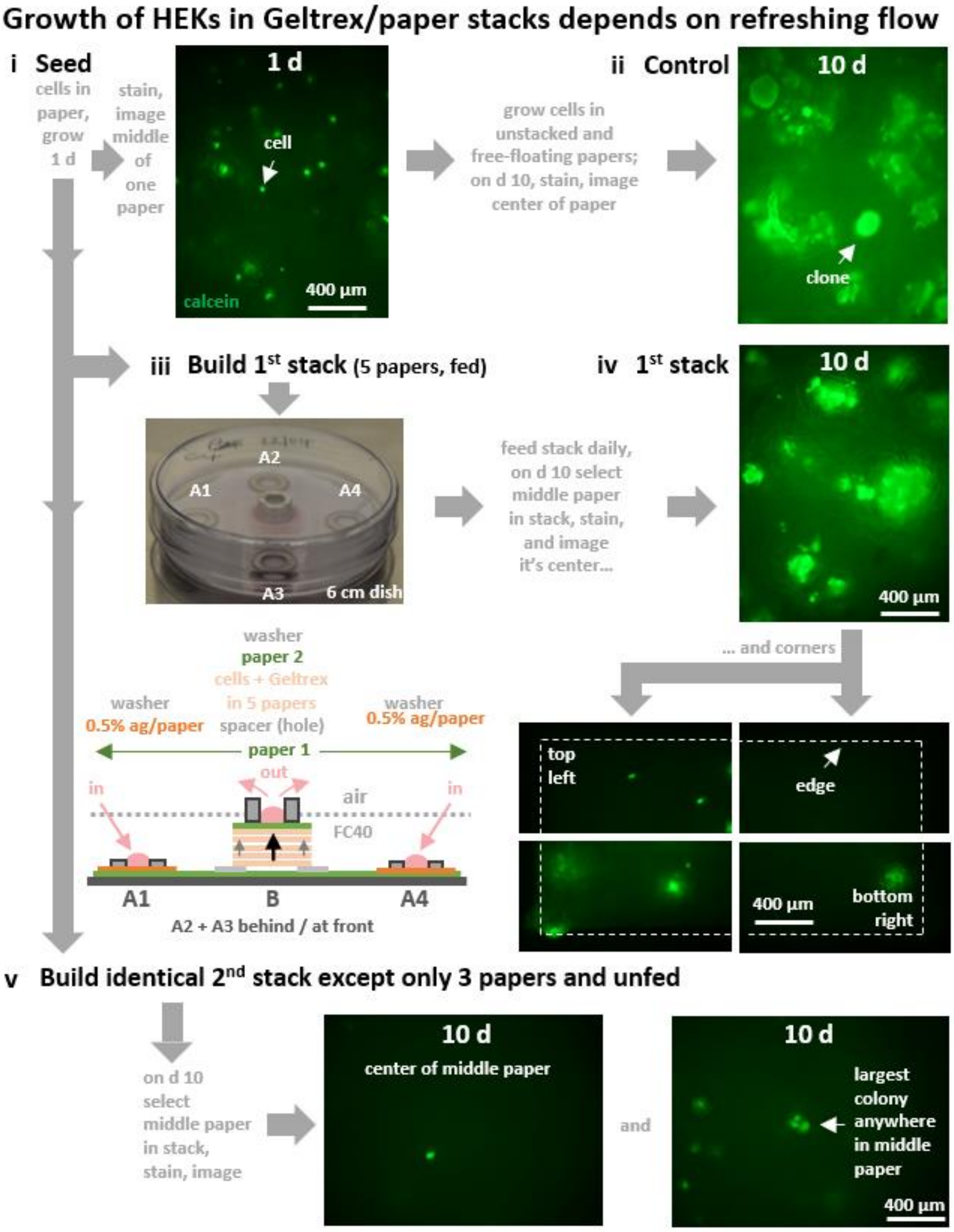

Fig. S7. Clonal growth of HEKs in filter-paper stacks depends on refreshing flows. Paper squares (10 x 10 mm Whatman No 113; thickness ~340 µm) containing cells are stained with calcein AM, and fluorescence images of different areas of squares collected. (**i**) Paper squares are dipped into ice-cold medium + 10% Geltrex containing HEKs (10^5/ml) so medium + cells wick into papers, and squares immediately dropped into medium (37°C) to set the gel. Cells now in unstacked squares are grown for 1 d, stained, and the center of a square imaged. Bright spots mark single or newly-divided cell pairs (arrow marks a single cell). (**ii**) As controls, unstacked and squares immersed in medium are grown until d 10, and cells stained and imaged; fluorescent colonies enlarge over time, fed by diffusion (arrow marks a colony). (**iii**) A circuit with a stack of 5 squares is also built on d 1. Image: circuit with its central 5-paper stack surrounded by 4 input reservoirs (A1-A4). Cartoon: medium is pipetted into inputs A1-A4 so differences in hydrostatic pressure

Biology Open • Supplementary information

drive flow down through agarose pores in flow regulators, laterally through the octagonal filter-paper base to stack B, up through the hole in a spacer, past cells in 5 squares, and into the output cap. As the spacer is relatively impermeable, flow upwards is higher through the middle of the stack (black arrow) compared to the sides (grey arrows). (**iv**) This circuit with 5 papers is fed daily by pipetting medium into A1-A4; on d 10, the square in the middle of the stack is removed, stained, and imaged. Fluorescent colonies develop much as in the control in the middle of this paper (top image); however, those in the 4 corners in flow-poor zones above the spacer are smaller (bottom 4 images). (**v**) An identical stack is also built on d 1 except the stack contains only 3 papers; this stack is not fed. On d 10, the middle paper in the stack of three is removed, stained, and imaged. Few colonies are seen in the center of this middle paper (left), and the largest colony anywhere in it is shown (right).

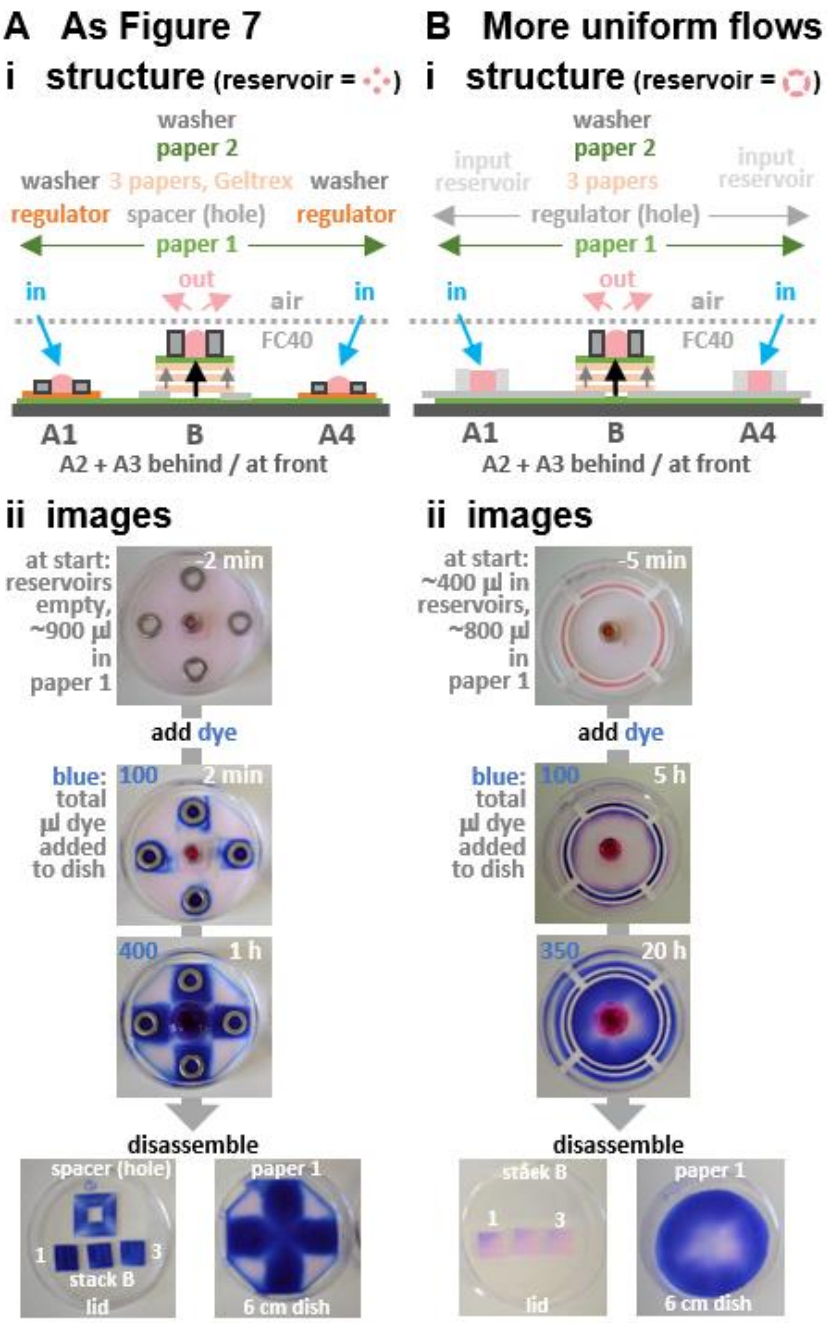

Biology Open • Supplementary information

Fig. S8. Flows through two circuits; the 1st is like that in Figure 7, the 2nd yields slower and more uniform flows (60 mm dishes; no cells present in stack B, experiments conducted at ~20 C).

A. Circuit as in Figure 7.

(**i**) Circuit structure.

(**ii**) Images are collected after adding medium + blue dye (in 4 x 25 µl aliquots) to reservoirs A1-A4 at t = 0, 15, 30 and 45 min (blue: accumulated volume added). After 2 min, two input reservoirs are empty (not visible), some dye is streaking over surfaces to stack B, and the cap is filling with dye-free medium initially present in paper 1. After 1 h, dye fills the cap, and some passes around edges of octagonal paper 1. After disassembly, Geltrex-containing papers (1-3 from stack bottom to top) contain dye. Results are consistent with rapid flow after each input.

B. A circuit yielding slower and more uniform flows.

(**i**) Circuit structure. A circular 3D-printed reservoir (1.5 mm wide) is divided by axial struts into 4 quadrants – sub-reservoirs A1-A4; it sits on a hydrophobic-paper disc that acts as the sole regulator for all sub-reservoirs (this disc has a central hole). This disc sits, in turn, on disc-shaped paper 1. We reasoned that rapid flows over/around regulators and paper 1 in (A) follow surface discontinuities and/or emerge through larger-than-average surface pores where Laplace pressures are smaller. If so, smoother papers (with smaller pores + higher Laplace pressures) should reduce such flows. Here the flow regulator and paper 1 are made of smoother papers; paper 1 is also thinner with smaller pores, so flow should be further limited. Stack B is as in (A), except the 3 papers lack dilute Geltrex.

(**ii**) Images. Each of the 4 reservoirs initially contain 100 µl medium (at t = -5 min). At t = 0, the washer on stack B is filled until medium is level with its top, and 25 µl blue dye pipetted into quadrants A1-A4. After 5 h, some dye is moving towards stack B, where the cap is filling with dye-free medium from paper 1. 140 µl is now removed from the cap so the top-most point is level with the top of the washer (indicating a flow rate of 140 µl/5 h). More dye (150 µl) is now added to A1-A4. After 20 h, dye is just reaching the center, and the cap is refilling (at ~400 µl/15 h). After disassembly, just the top left-hand edges of papers from stack B (1-3 from bottom to top) contain some dye.

We draw various conclusions by comparing results from (A) and (B): flow is substantially slower through the 2nd circuit without detectable streaky flow (consistent with smoother papers limiting surface flow). We would like to improve circuit design further to ensure even slower and more uniform flow (and so more convenient daily or weekly inputting), and to reduce expense (by reducing the volume of medium held in paper 1).

Biology Open • Supplementary information

Table S1. A wish list of requirements for building organs from different cell types in vitro.

		Requirement		Reference
**1**		Cells in diffusional range (<200 µm) of refreshing flows		Crick, 1970; Sharpe, 2019
**2**		Individual cell types grown in appropriate layers		Goodwin & Nelson, 2021
**3**		Aqueous connectivity between layers allows inter-layer signaling		Goodwin & Nelson, 2021
**4**		Cells in one layer able to move over/through cells in another		Goodwin & Nelson, 2021
**5**		Layer-specific ECMs with pores of diameter ~200-800 µm		Caliari & Burdick, 2016
**6**		Volumes small (reagent savings), so tessellate modules → larger organoids		
**7**		Rapid prototyping (e.g., swap modules in/out)		
**8**		Accessible to bioscientists (e.g., use standard dishes, incubators, etc.)		

Biology Open • Supplementary information
